# Modeling a Snap-Action, Variable-Delay Switch Controlling Extrinsic Cell Death

**DOI:** 10.1371/journal.pbio.0060299

**Published:** 2008-12-02

**Authors:** John G Albeck, John M Burke, Sabrina L Spencer, Douglas A Lauffenburger, Peter K Sorger

**Affiliations:** 1 Department of Systems Biology, Harvard Medical School, Boston, Massachusetts, United States of America; 2 Biological Engineering Department, Massachusetts Institute of Technology, Cambridge, Massachusetts, United States of America; 3 Computational and Systems Biology Initiative, Massachusetts Institute of Technology, Cambridge, Massachusetts, United States of America; Johns Hopkins University, United States of America

## Abstract

When exposed to tumor necrosis factor (TNF) or TNF-related apoptosis-inducing ligand (TRAIL), a closely related death ligand and investigational therapeutic, cells enter a protracted period of variable duration in which only upstream initiator caspases are active. A subsequent and sudden transition marks activation of the downstream effector caspases that rapidly dismantle the cell. Thus, extrinsic apoptosis is controlled by an unusual variable-delay, snap-action switch that enforces an unambiguous choice between life and death. To understand how the extrinsic apoptosis switch functions in quantitative terms, we constructed a mathematical model based on a mass-action representation of known reaction pathways. The model was trained against experimental data obtained by live-cell imaging, flow cytometry, and immunoblotting of cells perturbed by protein depletion and overexpression. The trained model accurately reproduces the behavior of normal and perturbed cells exposed to TRAIL, making it possible to study switching mechanisms in detail. Model analysis shows, and experiments confirm, that the duration of the delay prior to effector caspase activation is determined by initiator caspase-8 activity and the rates of other reactions lying immediately downstream of the TRAIL receptor. Sudden activation of effector caspases is achieved downstream by reactions involved in permeabilization of the mitochondrial membrane and relocalization of proteins such as Smac. We find that the pattern of interactions among Bcl-2 family members, the partitioning of Smac from its binding partner XIAP, and the mechanics of pore assembly are all critical for snap-action control.

## Author Summary

In higher eukaryotes, tissue development and homeostasis involves a subtle balance between rates of cell birth and death. Cell death (apoptosis) is triggered by activation of caspases, specialized enzymes that digest essential cellular constituents and trigger degradation of genomic DNA. Under normal circumstances receptor-dependent cell death is very tightly repressed, but it is irreversibly induced upon receipt of an appropriate signal. Mutations that interfere with this all-or-none control contribute to developmental abnormalities, autoimmune disease, and cancer. The biochemical properties of most apoptotic proteins are quite well understood, but it is unclear how these proteins work together. By combining live-cell microscopy, genetic perturbation, and mathematical modeling, we seek quantitative insight into cell death with a focus on network dynamics and control. We find that cells vary dramatically in the time between receipt of an apoptotic signal and the commitment to death. This variability arises from cell-to-cell differences in the activities of receptor-proximal biochemical reactions. Rapid all-or-none progress from commitment to actual death is achieved downstream by pro-apoptotic proteins found in the mitochondrial membrane. Our work provides a quantitative picture of apoptosis that advances understanding of oncogenic mechanisms and should eventually assist in the development of pro-apoptotic cancer therapies.

## Abstract 3

A combination of single-cell experiments and mathematical modeling reveals the mechanisms underlying all-or-none caspase activation during receptor-induced apoptosis.

## Introduction

Apoptosis is essential for the development of multicellular organisms but is misregulated in diseases as diverse as cancer and autoimmunity [1,2]. Activation of the potent effector caspases (caspases-3 and −7; hereafter C3 and C7), the hallmark of apoptosis, is triggered via the intrinsic cell death pathway by intracellular events such as DNA damage and oxidative stress, and via the extrinsic cell death pathway by extracellular stimuli such as TNF (tumor necrosis factor) and TRAIL (TNF-related apoptosis-inducing ligand) [3]. C3 and C7 directly degrade the proteome and, by activating DNAses, also dismantle the chromosomes of cells committed to die [4]. Caspase activation represents an irreversible change in cell fate and is consequently regulated at multiple levels, including assembly of complexes on death receptors [5], binding of pro- and anti-apoptotic members of the Bcl-2 family to each other in cytosolic and mitochondrial compartments [6,7], mitochondria-to-cytosol translocation of Smac and cytochrome c (CyC) [8–10], and direct repression of caspases by inhibitor of apoptosis proteins (IAPs) [11]. Studies of extrinsic apoptosis at the single-cell level reveal a long and variable delay prior to effector caspase activation but rapid and sudden progression to substrate cleavage once activation has begun [12,13], a behavior that we term “variable-delay, snap-action” switching. Failure in snap-action switching generates an indeterminate physiological state and sublethal cellular damage that may predispose cells to genomic instability [14,15].

A variety of kinetic models of apoptotic cell death have been published to date [16–26], most of which are based on coupled systems of differential equations. These models incorporate different mechanisms for achieving all-or-none caspase activation, including positive feedback via caspase-8 (C8) [17,20] or caspase-9 (C9) [22,23], and ultra-sensitivity in C9 activation [20]. Most models also focus on subsets of reactions, such as those stimulated directly by death receptors [17] or those downstream of mitochondrial outer membrane permeabilization (MOMP) [23] rather than on the interplay between upstream and downstream reactions. Moreover, most studies rely on previously published data rather than cycles of model-based hypothesis generation and experimental test. In this paper we attempt to overcome these limitations by including upstream and downstream reactions in a single model and by tightly coupling modeling and experimentation. The ordinary differential equation (ODE)-based model of C3 regulation we describe is based on mass action kinetics and has been trained against population-based and single-cell data obtained from cells perturbed by RNA interference (RNAi) and protein overexpression. We establish a role for C8 and its substrates in pre-MOMP delay and explore how competition among pro- and anti-apoptotic Bcl-2 family members determines when MOMP occurs. We also examine a series of alternative topologies for reactions involving Bcl-2-like proteins and uncover a subtle interplay between protein compartmentalization, translocation, and multimerization in the regulation of snap-action switching.

## Results

### Modeling Extrinsic Cell Death Pathways

**Figure 1:**
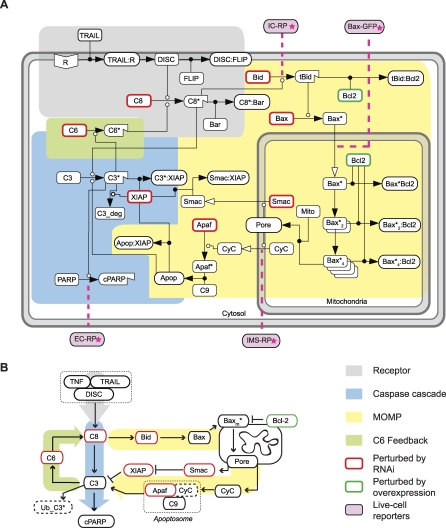
Process Diagram of the Death Receptor Network Modeled in This Study (A) The convention of Kitano et al. [94] is followed. The major features of the network are highlighted by color: gray, receptor module; blue, direct caspase cascade; green, positive feedback loop; yellow, mitochondrial feed-forward loop. (B) A condensed alternate representation of the network.

A mathematical model of proteins known to regulate C3 during extrinsic apoptosis was constructed on the basis of mass-action kinetics, with elementary reactions represented as ODEs (Figure 1; Protocol S1; Tables S1–S6). All biochemical transformations were represented as unimolecular or bimolecular reactions, and rate laws were therefore expressed as *r* = *k* × [*A*], for a reaction involving one copy of protein *A*, *r* = *k* × [*A*] × [*B*], for a bimolecular reaction of *A* and *B*, or *r* = *k* × [*A*] × [*A*], for dimerization of *A* (Tables S2 and S6). Transport between cellular compartments was also modeled as an elementary unimolecular reaction, and the assembly of multiprotein complexes as a series of bimolecular reactions. Because no complex algebraic forms such as Hill functions were used in our model, ultrasensitivity and other nonlinear behaviors arise from interactions among simple elementary reactions rather than the properties of higher-order equations. Where possible, estimates for model parameters (rates and initial protein concentrations) were obtained from the literature (Tables S4 and S5). In the absence of such information, kinetic rate constants and initial conditions were set to intermediate values within a physically plausible range and then fitted so as to optimize model performance [27].

Well-substantiated biochemical reactions comprising four interacting cell death subcircuits were included in the “extrinsic apoptosis reaction model” described here (EARM v1.0), but some regulatory processes were simplified or omitted (Figure 1). EARM v1.0 contains 58 species corresponding to 18 gene products having nonzero initial conditions and 40 additional species representing complexed, cleaved, or differentially localized forms of the initial species, which interact via 28 reactions described by 70 nonzero rate constants (including forward, reverse, and *k*_cat_ rates for each reaction; Tables S1 and S2). The four subcircuits in EARM v1.0 comprise (i) a lumped-parameter representation of receptor binding by TNF or TRAIL and the subsequent activation of pro-C8 by receptor-bound death-inducing signaling complexes (DISC) to form C8* (Figure 1, gray); (ii) an enzyme cascade in which C8* directly cleaves C3 [28] to form active C3*, which can cleave effector caspase substrates (a process represented in our model by cleavage of PARP to form cPARP [29]) but not when bound to XIAP (X-linked IAP [11] Figure 1, blue); (iii) a mitochondrial feed-forward pathway in which C8* cleaves Bid (into tBid) [30] to activate Bax (to Bax*) and promote formation of pores in the mitochondrial membrane through which CyC [10] and Smac [8,9] translocate into the cytosol following MOMP; cytosolic CyC then binds Apaf-1 and C9 to form the apoptosome (which also cleaves pro-C3 [31,32]), and Smac neutralizes XIAP [33–36], thereby de-inhibiting C3* (Figure 1, yellow); (iv) a positive feedback loop in which pro-caspase-6 (pro-C6) is cleaved by C3* to form C6* [37], which then activates additional pro-C8 (Figure 1, green) [38,39].

EARM v1.0 aims to be reasonably complete with respect to biochemical mechanism, but three simplifications were made to reduce the number of species and free parameters. First, the details of DISC and apoptosome assembly, both of which involve multiple copies of several protein species [40,41], were omitted in favor of simplified “lumped parameter” representations. Second, protein synthesis was omitted because all experiments were performed in the presence of cycloheximide (which is commonly used to sensitize cells to the action of TNF [42], but which, in our experiments, also simplifies modeling by eliminating source terms). Third, proteins with similar biochemical activities were represented by a single species: C8 and caspase-10 (C10) by C8 alone; C3 and C7 by C3 alone; and the Bcl-2-like family of proteins by three prototypical examples: Bid, a pro-apoptotic “activator,” Bcl-2, an apoptosis inhibitor, and Bax, a pore-forming protein. We are aware of controversy regarding the precise mechanism by which Bcl-2-like proteins regulate MOMP and have implemented the simplest form of “direct activation” [43]. Further research will be required to determine whether kinetic modeling can help to distinguish this scheme from alternative “indirect activation” hypotheses [25,44].

### Experimental Determination of C3 Activation Dynamics

To gather data for model training, we first sought to establish precise dose-response relationships for death ligands in HeLa cells, which are widely used in cell death studies and a robust biological setting in which to combine RNAi and single-cell imaging [45–48]. HeLa cells were exposed to TRAIL over a 500-fold range of concentrations spanning roughly physiological to saturating. Cell death was monitored by live-cell microscopy using either of two Förster resonance energy transfer (FRET)-based reporter proteins whose fluorescence changes upon cleavage (effector or initiator caspase reporter proteins, EC-RP and IC-RP) and a reporter for mitochondrial outer membrane permeabilization (mitochondrial intermembrane space reporter protein [IMS-RP]) whose cytosolic translocation mimics that of Smac and CyC [15].

**Figure 2:**
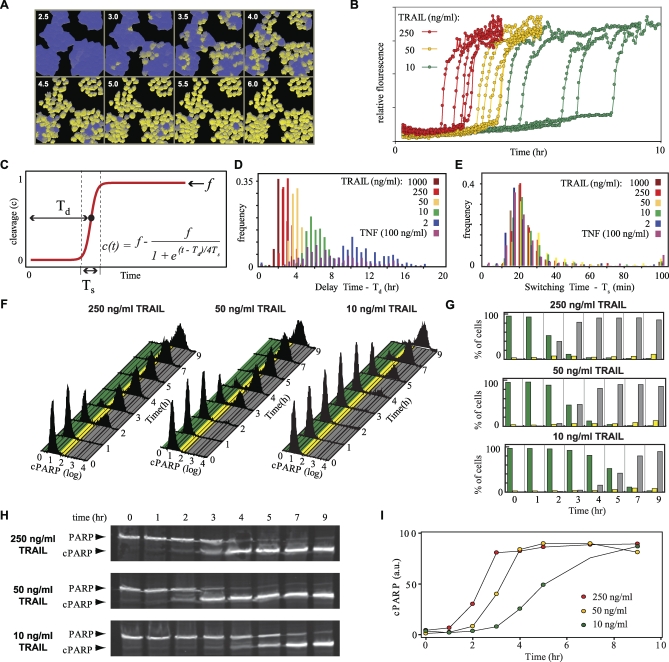
Dynamics of Caspase Activation in Cells Treated with TNF or TRAIL (A) Time-lapse images of HeLa cells expressing EC-RP treated with 50 ng/ml TRAIL. Imaging was performed at 465 nm and 535 nm; cleavage of EC-RP caused a shift in the 465/535 ratio that was pseudo-colored as a shift from blue to yellow. Inset numbers indicate the time in hours after treatment with TRAIL. (B) Time courses of EC-RP cleavage in individual HeLa cells treated with 250, 50, or 10 ng/ml TRAIL; five cells are shown for each dose. Cell-to-cell variation in dynamics for a single dose is observed reproducibly and is not a consequence of imprecision in the assay. (C) Idealized single-cell time course for EC-RP cleavage, showing relationships among T_d_ (delay time), T_s_ (switch time), and *f* (fraction substrate cleaved). (D, E) Frequency distributions for T_d_ (D) and T_s_ (E) determined by live-cell microscopy in EC-RP-expressing HeLa cells treated with varying concentrations of death ligand (*n* > 100 for each condition); binning intervals were 30 min (E), or 5 min (F). (F) Flow cytometry of PARP cleavage, as assayed with an antibody selective for PARP cleaved at Asp214 by C3 and C7, in HeLa cells treated with 10 to 250 ng/ml TRAIL for 1–9 h as indicated. Colored regions underlying the histogram represent intervals used to discretize data into PARP cleavage levels of <5% (green), 5%–25% (yellow), or >25% (gray; percentages are relative to the median fluorescence intensity of the fully positive population). (G) Discretized flow cytometry data. The fraction of cells having low, intermediate, or high PARP cleavage is color-coded to match the intervals in (F). (H) Immunoblot analysis of PARP cleavage in HeLa cells treated with 10 to 250 ng/ml TRAIL. (I) Quantitation of the cleaved 89 kDa form of PARP for blots shown in (H).

Following TRAIL exposure, C3* activity remained low, as measured by EC-RP fluorescence, for a period of time that varied widely from cell to cell before rising rapidly to a plateau, at which point cells died (Figure 2A and 2B) [15]. Prehn and colleagues [12] have shown that these dynamics have the general form:


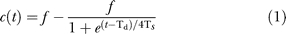


where *c(t)* is the amount of substrate cleaved at time *t*, *f* is the fraction cleaved at the end of the reaction, T_d_ is the delay period between TRAIL addition and half-maximal substrate cleavage (*c(t)=* 0.5 *f*), and T_s_ is the switching time between initial and complete effector substrate cleavage (the reciprocal of the slope at *t* = T_d_; Figure 2C). A fourth parameter, the time constant of the cleavage reaction T_c_ = T_s_ × *f*, provides a measure of switching time that is independent of the final amount of substrate cleaved. When live-cell data from ∼150 TRAIL-treated cells were parameterized using Equation 1, T_d_ varied from 1 to 15 h depending on ligand dose (Figure 2D and Table 1). TNF treatment, even at saturating concentrations of ligand, elicited a response that was even more heterogeneous with respect to T_d_. In contrast, T_s_ and *f* varied little from cell to cell regardless of ligand dose or identity (with mean values ranging from 19 to 27 min for T_s_; Figure 2E and Table 1). Thus, in accordance with previous studies [12], we find the extrinsic pathway of cell death to involve dose- and ligand-dependent variation in T_d_ concomitant with maintenance of T_s_ at a constant value.

**Table 1:**
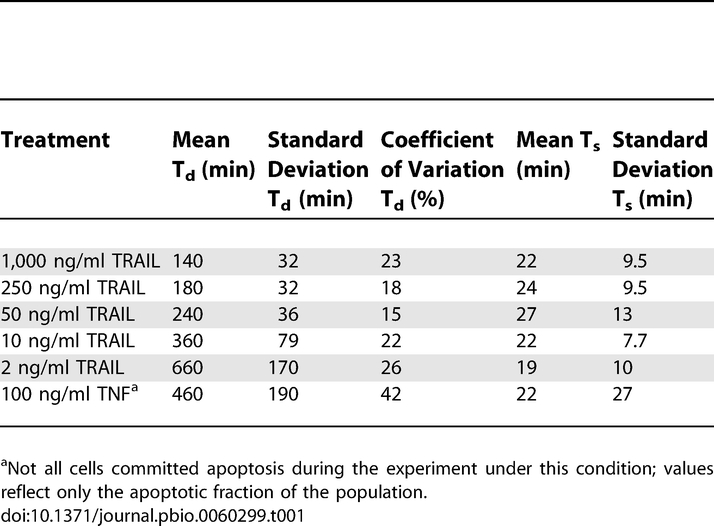
Values for Switching Parameters Determined by Live-Cell Microscopy

A caveat of using fluorescent reporter proteins to study apoptosis is that cleavage of synthetic substrates is not necessarily representative of endogenous substrate cleavage. Cleavage of the endogenous C3* substrate PARP was therefore monitored by flow cytometry of cells stained with antibodies specific for the cleaved form (cPARP) [49]. Over a range of TRAIL concentrations, a bimodal distribution in fluorescence intensities was observed in which antibody-nonreactive cells became less abundant with time, while antibody-reactive cells became more abundant (Figure 2F). Similar data were also obtained for the C3* substrate cytokeratin (Figure S1). To quantify these changes, fluorescence signals were discretized into levels corresponding to no detectable PARP cleavage (Figure 2G, green bars), maximal cleavage (grey bars), and cleavage of a subset of the PARP in each cell (yellow bars; as opposed to complete PARP cleavage in a subset of cells). Regardless of ligand dose or identity, <5% of cells scored as having partially cleaved PARP, as expected for cells that undergo a sudden transition to from life to death [50]. Thus, flow cytometry shows cleavage of endogenous C3* substrates to be rapid and complete at the single-cell level. To ascertain whether maximal fluorescence as measured by flow cytometry or FRET actually corresponds to complete cleavage, we monitored PARP levels and molecular weight by immunoblotting. Conversion of >80% of 112 kDa full-length PARP into 89 kDa cPARP was observed by 9 h at all doses of TRAIL (Figure 2H and 2I), and we therefore conclude that *f* ≈ 1.0 under our experimental conditions.

### Merging Data from Single-Cell and Population-Based Measurements

**Figure 3:**
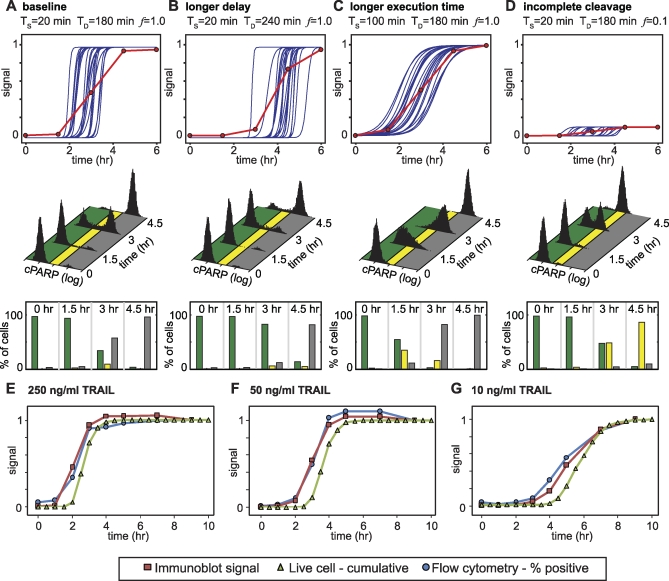
Simulation-Based Approach to Data Fusion for Three Different Measures of Caspase Activity (A, B, C, D) Simulated live-cell (top, twenty individual cells shown as blue lines), immunoblot (top, red lines), raw flow cytometry (middle, computed from 10,000 simulated live-cell profiles), and discretized flow cytometry data (bottom) for four different sets of switching parameters: (A) Baseline, corresponding to 250 ng/ml TRAIL; (B) an increase in mean T_d_ to 240 min. (C) An increase in T_s_ to 100 min (D) or a decrease in *f* to 0.1. (E, F, G) Concordance of data on EC substrate cleavage as obtained from immunoblot, flow cytometry, and live-cell assays in HeLa cells treated with 250 ng/ml (E), 50 ng/ml (F), or 10 ng/ml (G) TRAIL. Values for immunoblots are derived from quantitating the cleaved 89 kDa form of PARP by immunoblot; flow cytometry values represent the percentage of cPARP-positive cells computed as described in the legend of Figure 2, and live cell data were computed as the cumulative percentages of dead cells for populations of >150 cells. To facilitate comparisons, all three signals were normalized to their final value.

To merge data obtained by imaging, flow cytometry, and immunoblotting and thereby quantify the mean and variance of T_s_, T_d_, and *f*, we simulated the experimental procedures involved (Protocol S2). Synthetic live-cell data were generated for 10,000 idealized TRAIL-treated cells assuming snap-action, variable-delay caspase activation as per Equation 1 with *f* = 1.0 and normal distributions for T_s_ = 20 ± 10 min and T_d_ = 180 ± 40 min (values were based on live-cell data for cells treated with 250 ng/ml; ranges represent standard deviations)*.* 10,000 synthetic live-cell trajectories were then averaged at discrete points in time to generate synthetic immunoblot data (Figure 3A). Synthetic flow cytometry data were generated from single-cell trajectories by computing distributions of caspase substrate cleavage at fixed points in time. Empirically derived values for background fluorescence and measurement noise were added, the data were discretized into 1,024 bins (corresponding to 10-bit detection), and results were plotted on a log-linear scale (Figure 3A). Varying the mean values of T_s_, T_d_, or *f* in the synthetic trajectories revealed that: (i) when T_d_ alone increases, cPARP levels remain bimodal when monitored by flow cytometry (Figure 3B); (ii) when T_s_ is increased with *f* constant, bimodality is lost and cells having intermediate levels of cPARP accumulate at short but not long times (because *f* → 1.0 as *t* → ∞) (Figure 3C, yellow bars); (iii) when *f* decreases with or without changes in T_d_ and T_s,_ many cells exhibit partial PARP cleavage even at long times (Figure 3D). Analogous inverse procedures make it possible to compare live-cell imaging, flow cytometry, and immunoblotting data quantitatively. In addition, they emphasize that the three methods differ in their ability to estimate mean values and distributions for T_s_, T_d_, or *f* (Table 2). Flow cytometry, for example, yields indirect measures of the mean and variance of T_s_, T_d_, and relative values for *f* at discrete points in time, while immunoblotting provides a good estimate of the average absolute value of *f.* Moreover, one measurement—suitably processed—usually adds information to another; determination of *f* by immunoblotting, for example, enables calibration of flow cytometry and live-cell microscopy values.

**Table 2:**
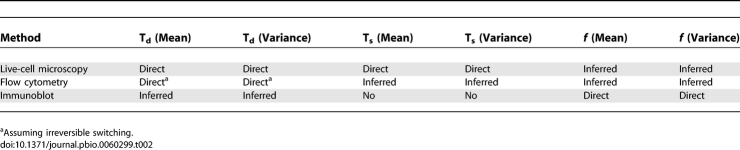
Experimental Determinability of Switching Parameters

When experimental live cell, flow cytometry, and immunoblot data from HeLa cells exposed to TRAIL at three different doses were merged, excellent quantitative agreement in death dynamics was observed (Figure 3E–3G). This confirms that EC-RP and IC-RP are effective reporters of endogenous caspase substrate cleavage and that an increase in TRAIL dose from 2 ng/ml to 1,000 ng/ml causes T_d_ to vary 4- to 5-fold even as T_s_ and *f* remain constant at ∼20 ± 10 min and 1.0, respectively. Quantitative models of apoptosis must account for the overall efficiency of this process, the dose-dependence and length of T_d_, the rapidity of T_s_, and the independence of T_s_ and T_d_.

### Linking Models and Experiment Via Perturbation

**Figure 4:**
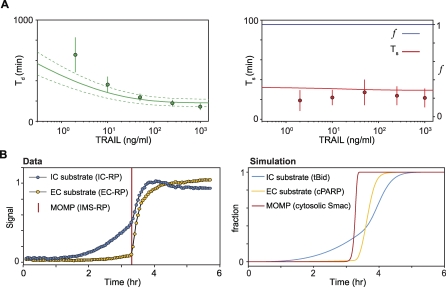
Training Data Derived from Live-Cell Microscopy (A) Simulation of T_d_ (left) or T_s_ and *f* (right) as a function of TRAIL dose (lines) alongside corresponding experimental values (points with error bars indicating standard deviations). For predicted values of T_d_, an envelope of constant coefficient of variation is shown, as estimated from experimental data (CV ≈ 20%); the source of variation is not known. (B) Composite plot of IC-RP and EC-RP cleavage for >50 cells treated with 50 ng/ml TRAIL in the presence of CHX and aligned by the average time of MOMP (left panel) and model-based simulation of the corresponding species (right panel). Data in the left panel were originally reported elsewhere [15].

**Figure 5:**
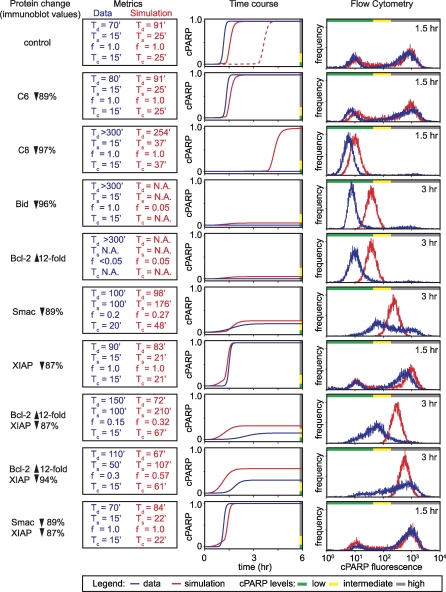
Training the Model on Network-Wide Perturbations First column: perturbation values measured by immunoblot (see Figure S2). Second column: comparison of EARM v1.0-simulated and experimentally derived values for T_s_, T_d_, *f*, and T_c_. Simulated values were computed by EARM v1.0 with the perturbation conditions indicated in the first column. Experimental values were derived by fitting the simFACS data model to discretized flow cytometry data (See Materials and Methods and Protocol S2 for details). Third column: comparison of EARM v1.0-simulated cPARP cleavage (red) to time courses derived from flow cytometry using a data model (blue). Blue curves were produced by Equation (1) parameterized with the experimental T_s_, T_d_, and *f* values shown in the second column. The dashed line in the control condition shows simulation under non-siRNA conditions; all other simulations were performed under siRNA conditions (see “Linking Models and Experiment Via Perturbation” for details). Fourth column: comparison of experimental (blue) and predicted (red) flow cytometry plots at the indicated time points (see Figure S3 for comparisons of all time points). Predicted flow cytometry data were produced by simFACS data simulation using the EARM v1.0-simulated values for T_s_, T_d_, and *f* shown in the second column. Raw data for several of the conditions shown were originally reported elsewhere [15].

Parameters in EARM v1.0 were manually adjusted to minimize the difference between simulated trajectories and experimental data including: (i) mean values for T_s_, T_d_, and *f* in cells exposed to a range of TRAIL concentrations (Figure 4A); (ii) composite live-cell time-courses of initiator and effector caspase activity and MOMP (Figure 4B); (iii) estimates of T_s_, T_d_, and *f* obtained by flow cytometry of TRAIL-treated cells perturbed by small interfering RNA (siRNA) or protein overexpression (Figures 5 and S3). To obtain composite time courses, ∼100 live-cell measurements of individual cells expressing IMS-RP and either IC-RP or EC-RP were aligned by the time of MOMP. As we have recently reported [15], gradual cleavage of IC-RP but not EC-RP was apparent during the pre-MOMP delay. MOMP occurred once the IC-RP signal rose to ∼30%–50% of its maximum value and EC-RP was then cleaved to near completion within 20–30 min (Figure 4B).

Data on perturbed cells were obtained by siRNA-mediated protein depletion (typically, three independent siRNA oligos were validated for each gene; Figures 5 and S2) or by protein overproduction in cells stably expressing cDNAs under the control of viral promoters. Simulating a reduction in the abundance of a species 5- to 20-fold often had a different outcome from simply eliminating the species. Thus, RNAi was modeled by adjusting the initial value of a species ([*X*]_0_ for species *X*) to the measured extent of protein depletion as determined by semiquantitative western blotting . Uniformity of depletion was established using flow cytometry (Figure S2). A subtlety that arose when linking models to data was a ∼100–150 min reduction in mean T_d_ following transfection of cells with nontargeting siRNA oligos. The reduction probably reflects siRNA-mediated induction of interferon-stimulated genes [51], which include the DR5 TRAIL receptor and C8 [52–54]. To account for this effect, cells transfected with targeting and nontargeting RNAi were modeled as having more TRAIL receptors (1 × 10^5^ versus 2 × 10^2^ per cell; Figure 5 and Table S5). Experimentally, targeting oligos were always compared in parallel to nontargeting controls.

Perturbation of the mitochondrial pathway (Figure 1, yellow circuit) by Bid depletion or Bcl-2 overexpression blocks cell death in response to TRAIL (Figure 5). To monitor the dynamics of the cytosolic reactions in the absence of the full mitochondrial feed-forward pathway, it is necessary to combine Bcl-2 overexpression with XIAP depletion [15]. Under these conditions, only a fraction of cells died in response to TRAIL, with T_s_ 3- to 5-fold longer than in unperturbed cells and C3 substrate cleavage incomplete (*f* = 0.15–0.3; Figure 5). The phenotype of Smac depletion was very similar, and both represent a highly undesirable state of “partial” cell death in which effector caspases only achieve sublethal levels [15]. These data demonstrate that efficient snap-action cleavage of effector caspase substrates absolutely requires the mitochondrial feed-forward pathway: the direct C8* → C3* cascade and C3* → C6* → C8* feedback loop are insufficient.

Reconciling dose-response, composite time-course, and perturbation data placed significant demands on EARM v1.0. Accommodating the conflicting requirements that XIAP be sufficiently abundant to fully block C3* in the pre-MOMP interval and that XIAP be efficiently sequestered by cytosolic Smac following MOMP involved careful adjustment of species concentrations and reaction rates, a fragility that implies the existence of additional as-yet unknown regulatory processes [15]. Nonetheless, simulating TRAIL treatment over a range of concentrations resulted in nearly constant T_s_ of ∼30 min, *f* = 1.0, and dose-dependent variation in T_d_, all of which are in good agreement with experimental data (Figure 4A). Simulated time-courses for Bid cleavage (representing cumulative C8* activity), Smac translocation (MOMP), and cPARP levels (representing cumulative C3* activity) also matched experimental observations closely (Figure 4B). Efforts to improve the goodness of fit between model and experiment, assess model identifiability, and quantify parametric uncertainty are ongoing but are complicated by cell-to-cell variability and the consequent necessity of finding best-fit distributions of initial conditions and rate parameters rather than single values. To address this challenge, new methods will be required, but recent singular perturbation analysis of a reduced version of the EARM v1.0 model nonetheless suggests that parameter values reported here (Tables S3 and S5) represent reasonable order-of-magnitude estimates (J.M. Burke and P.K. Sorger, unpublished data).

### Transition from a Graded to a Snap-Action Signal

**Figure 6:**
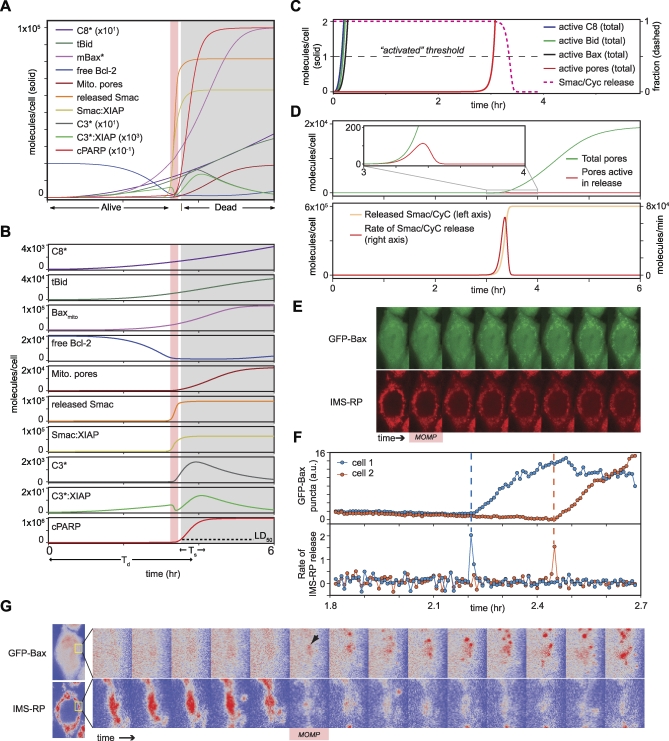
Prediction of the Transition from Graded to Switch-Like Kinetics For simplicity, positive feedback was omitted in all simulations by setting [C6]_0_ = 0; results with positive feedback produce highly similar conclusions and can be found in Figure S4. (A) Overlay of multiple species. To accommodate the wide range of concentrations, some species are scaled according to the values in parentheses in the caption. (B) The same data as in (A) except that each vertical axis is scaled independently to better depict the full dynamic range for each species. The pink vertical line denotes the duration of MOMP. The estimated LD_50_ for caspase activity (corresponding to ∼10% PARP cleavage) [15] is denoted by a dashed line in the cPARP plot; the gray shaded region denotes points in time after this lethal dose has been reached and cells are already destined to die. (C) Simulated time courses for C8*, tBid, Bax*, and mitochondrial pores as in (A) shown on a truncated vertical axis to display the times at which each species achieves a concentration of one molecule/cell. The full time course for mitochondrial Smac/CyC is shown as a dashed line on a full vertical axis (right) to show MOMP. (D) Top panel, simulation of the total number of MOMP pores as in (A) (green) in comparison to the subset of pores bound to Smac or CyC during the process of release (red). Bottom panel, simulation of released Smac/CyC as in (A) (pink) and the discrete-time derivative of the release reaction (red). (E) Live-cell imaging of IMS-RP (red) and GFP-Bax (green) in the same cell. Frames correspond to 30-s intervals, with the first frame of MOMP denoted by a pink box. Partial mitochondrial localization of IMS-RP following MOMP is an artifact of the overexpressed reporter; endogenous CyC is fully cytosolic at this point, as determined by immunofluorescence (not shown). (F) Quantitation of Bax pores (top) and rate of IMS-RP release (bottom) for two cells (orange curves correspond to the cell shown in [E]); puncta and rate of release were quantified as described in Materials and Methods. (G) Live-cell images for the cell in (E) shown at higher zoom and with pseudocoloring to highlight the temporal and spatial relationship between Bax puncta and IMS-RP release. Frames correspond to 30-s intervals; the magnified region (yellow box) encompasses the area of the cell in which both GFP-Bax puncta and IMS-RP release are first visible.

Differing dynamics of initiator caspase and effector caspase substrate cleavage raise the question of where in the extrinsic apoptosis pathway a steady and gradual increase in C8* activity is converted into a snap-action downstream signal. To address this issue, simulation was used to monitor each step in a “typical” cell (Figures 6A and S4), something that cannot be done experimentally. The binding of TRAIL to DR4/5 receptors results in gradual C8* accumulation, steady processing of Bid into tBid, and gradual accumulation of active Bax* (Figure 6B). In contrast, MOMP and the consequent release of Smac and CyC (which are identical in their release kinetics) from mitochondria via Bax*-containing pores is sudden and rapid, beginning ∼3 h post-TRAIL addition and reaching completion within ∼15 min.

Two complementary effects appear to account for the transition from graded C8* activation to snap-action Smac/CyC release: maintenance of a very tight “off” state during the pre-MOMP delay followed by creation of a low-impedance “on” state. When we examined the time at which each species reaches a threshold of one molecule/cell (an arbitrary level that is useful for illustration), C8*, tBid, and mitochondrial Bax* all exceeded this threshold within minutes of TRAIL addition, but Bax-containing pores (M*) remained below the threshold for hours until seconds before Smac/CyC translocation began (Figure 6C). Thus, pores are essentially absent during the long pre-MOMP delay. Simulation also shows that only about ∼100 pores (which require <15 min to form once assembly is initiated) are required to translocate >10^5^ molecules of Smac/CyC into the cytosol, because movement down a very steep concentration gradient is involved (Figure 6D). (This corresponds to the low-impedance “on” state.) Thus, the presence of very few active pores is massively amplified during Smac/CyC translocation, resulting in rapid depletion of protein from the intermembrane space and a switch-like response. Simulation also reveals that pore formation can continue for up to ∼2 h (assuming the cell does not lyse first; Figure 6D) so that it vastly exceeds what is required for Smac translocation. Ongoing pore formation appears to be an example of extreme overshoot: to induce efficient early translocation, the number of pores continues to rise well beyond what is required for Smac/CyC transport. Indeed, increasing the concentration of Smac in the model from 10^5^ to 10^9^ molecules/cell (an unrealistically large number) confirms that rapid release depends on excess pore capacity relative to the pool of proteins to be released (Figure S5).

### Predictions I and II: MOMP Is Complete by the Time Dying Cells Have Assembled Relatively Few Pores

Two testable predictions arise from the simulation of MOMP described above: (i) Smac release should begin nearly simultaneously with the formation of the first Bax-containing pores, and (ii) pore formation should continue long after Smac release is complete. To test these predictions, cells expressing green fluorescent protein (GFP)-Bax and IMS-RP were treated with TRAIL (50 ng/ml) and imaged at 60× resolution at 30-s intervals for 1 h before and after MOMP. The rate of release of IMS-RP was estimated using an edge-detection algorithm sensitive to the transition from clustered IMS-RP signals diagnostic of mitochondrial localization to diffuse signals diagnostic of cytosolic localization. In agreement with previous studies [55] and the assumptions in EARM v1.0, GFP-Bax had a diffuse cytosolic localization prior to MOMP but, in dying cells, formed bright puncta that colocalized with mitochondria (as marked by IMS-RP; Figure 6E). Moreover, GFP-Bax puncta appear to be identical to puncta detected by immunofluorescence microscopy (not shown), suggesting that GFP-Bax is representative of endogenous Bax. Appearance of the first GFP-Bax puncta coincided with IMS-RP translocation, which reached a maximal level within 1–2 frames (∼1 min), after which puncta continued to form for 20–30 min more, typically rising to >100 per cell by the time cells began to fragment (at which point further observation was unreliable; Figure 6F and 6G). Live-cell studies by others using similar methods have also demonstrated a close temporal link between initial formation of Bax puncta and MOMP (D.R. Green, personal correspondence). We often observed that the timing of MOMP varied with location in a cell, such that IMS-RP release from some mitochondria preceded release from other mitochondria by ∼1 frame (30 s; Figure 6G). In these cases, the first observable Bax punctum was associated with the earliest-releasing subset of mitochondria. A similar relationship was observed between aggregation of GFP-Bak (a second pore-forming protein involved in MOMP) and IMS-RP translocation, with the exception that GFP-Bak was found on the mitochondrial membrane prior to MOMP [56]. The first appearance of GFP-Bak puncta was coincident with IMS-RP translocation and puncta once again continued to form for >20 min thereafter (Figure S6). In contrast, GFP-Bcl-2 and GFP-Bcl-X_L_ exhibited diffuse mitochondrial localization throughout, with no apparent changes during MOMP (unpublished data). Bid-GFP remained in the cytosol before, during, and after MOMP, suggesting that tBid dissociates from Bax and Bak prior to pore formation (some Bid-GFP aggregates were visible once membrane blebbing and cell shrinkage had begun but only long after MOMP was complete; unpublished data).

The precise relationship between functional translocation pores and visible GFP-Bak/Bax puncta is not known, and puncta visible by live-cell imaging certainly contain more than the 4–8 Bax or Bak subunits thought to comprise functional pores [56]. However, the close temporal and spatial association between puncta and IMS-RP release (particularly at different locations in a single cell) implies that puncta may be clusters of pores. Regardless, these experiments clearly confirm our two model based predictions (i) that cytosolic translocation of mitochondrial intermembrane proteins is complete by the time a relatively small number of pores have formed and (ii) that pores continue to form long afterward. Modeling also provides a possible explanation for this latter phenomenon: overshoot in pore forming reactions guarantees that the release of intermembrane proteins is sudden and complete regardless of variations in the rate of initial pore formation.

### Prediction III: Snap-Action Switching Does Not Require Feedback

**Figure 7:**
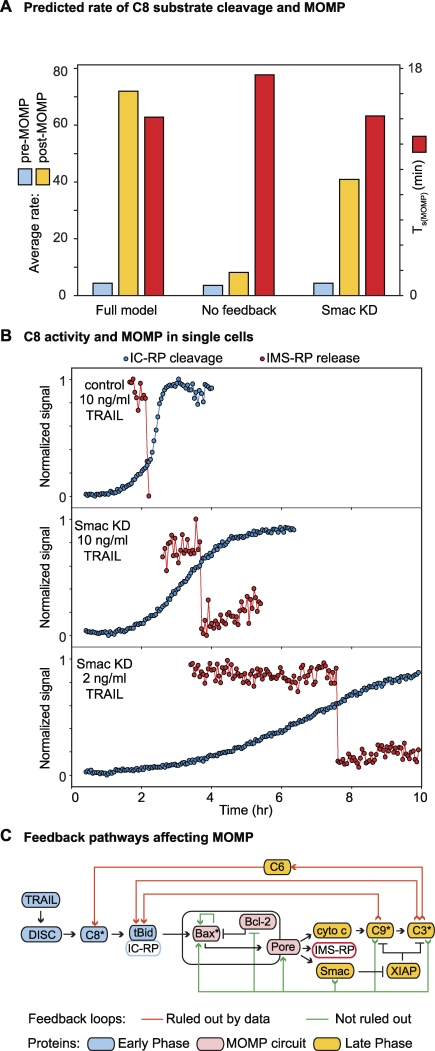
Rapid MOMP Independent of the Rapid Phase of Initiator Activity (A) Simulated values for the average rate of C8 substrate cleavage during the early (pre-MOMP) and late (post-MOMP) phases (left axis) and T_s_ of MOMP (right axis). Simulations were performed under conditions corresponding to stimulation by 2 ng/ml TRAIL. (B) Live-cell measurements of IC-RP cleavage and IMS-RP release in control or Smac-depleted cells treated with 10 or 2 ng/ml TRAIL. IC-RP and IMS-RP signals were normalized individually to allow comparison on the same axes. (C) Schematic diagram of potential feedback pathways regulating MOMP. The data shown in (B) are inconsistent with snap-action behavior resulting from feedback loops that act upstream of Bid cleavage (red arrows) but do not rule out the possibility that snap-action may arise from feedback loops that act downstream of Bid (green arrows). Some of the depicted loops are hypothetical and are shown for logical completeness.

It is an open question whether feedback from processes downstream of Smac/CyC translocation is necessary to ensure rapid all-or-none induction of MOMP. The existence of feedback has been proposed previously [37,39] and is likely to account for the biphasic cleavage of IC-RP following exposure of cells to TRAIL (Figure 4B). Previous analysis shows that slow pre-MOMP cleavage is entirely C8* dependent [15], but the rapid post-MOMP phase probably involves one or more feedback processes including (i) cleavage by C3* itself, (ii) cleavage by C8*, whose levels are expected to rise rapidly as a consequence of the C3* → C6* → C8* feedback loop, and (iii) cleavage by C9, an initiator caspase activated by CyC translocation. In simulation, inhibition of these feedback loops eliminates the second, rapid phase of IC-RP cleavage but has little effect on the dynamics of MOMP (Figure 7A). Modeling predicts that one simple way to attenuate feedback experimentally is depletion of Smac, since failure to inactivate XIAP blocks all three feedback loops (Figure 7A). We observed Smac-targeting siRNA oligonucleotides to eliminate the rapid phase of IC-RP cleavage, confirming the prediction that feedback from downstream to upstream processes had been inhibited (Figure 7B). Nonetheless, the kinetics of IMS-RP release were unaltered over a wide range of TRAIL concentrations. Most strikingly, when Smac-depleted cells were exposed to very low concentrations of TRAIL (2 ng/ml), IC-RP was processed very slowly over a 10-h period, but IMS-RP translocation was as rapid as in unperturbed cells in which IC-RP cleavage was ∼5-fold faster (Figure 7B, bottom panel). Thus, in agreement with simulation, rapid induction of MOMP does not appear to require any of the feedback loops that impact initiator caspase substrate cleavage. These include feedback-mediated C8 activation [39] and cleavage of Bid by C3* [57]. The data do not exclude a role for feedback acting downstream of tBid (such as self-activation of Bax [58]), but on the basis of model analysis, we speculate that feedback—if it exists—is not actually necessary for MOMP to achieve its snap-action character in HeLa cells (Figure 7C).

### Prediction IV: Dose-Dependent Activation of C8* Controls the Duration of T_d_

**Figure 8:**
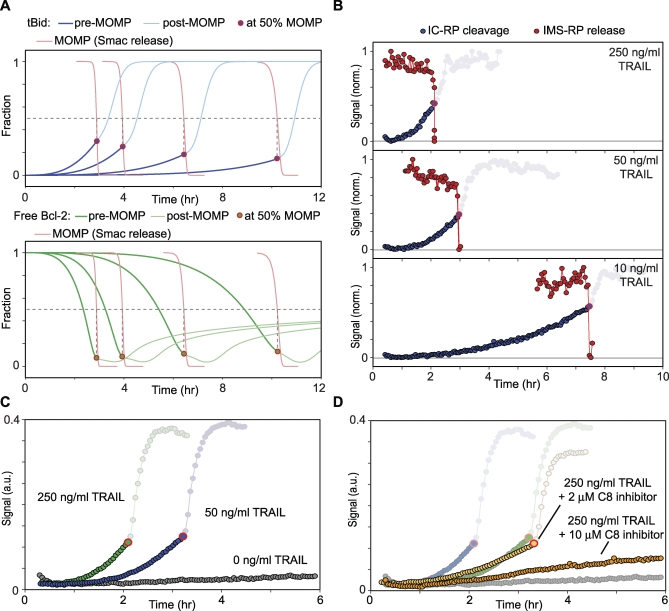
Control of T_d_ by C8 (A) Simulation of C8 substrate cleavage (tBid, top) and free mitochondrial Bcl-2 (bottom) in comparison to Smac/CyC release at 4 TRAIL concentrations (spanning approximately 0.2 to 200 ng/ml). Red circles indicate the level of tBid or Bcl-2 when Smac/CyC release is 50% complete. (B) Live-cell measurement of IC-RP cleavage and IMS-RP release in individual cells stimulated with 250, 50, or 10 ng/ml TRAIL. IC-RP and IMS-RP signals were normalized individually to allow comparison on the same axes. Circles outlined in red indicate the IC-RP signal at the time of IMS-RP release. (C) Averaged IC-RP cleavage for ten to 50 HeLa cells treated with 0 (black line), 50 (blue), or 250 (red) ng/ml TRAIL (as indicated) in the presence of cycloheximide. At each dose of TRAIL, time-courses were aligned by the average time of MOMP (indicated by symbols with red outlines). (D) Average IC-RP cleavage in 50 Hela cells treated with 250 ng/ml TRAIL in the presence of 10 or 2 μM C8 inhibitor. Time courses for cells treated with 2 μM inhibitor were aligned at the average time of MOMP. Average time courses from (C) are shown for comparison in light blue, green, and gray. In (C) and (D), MOMP did not occur in cells not treated with TRAIL or treated with TRAIL in the presence of 10 μM inhibitor, and time courses were aligned by the time of TRAIL treatment

Modeling suggests that the dose-dependence of T_d_ is determined by the time required to saturate Bcl-2 with Bax* and thereby generate Bax* active in pore-formation. This, in turn, depends on the rate at which tBid is generated by C8* (Figure 8A) (and by other factors as well). Thus, we predict that T_d_ is controlled by dose-dependent changes in C8* activity. Consistent with this hypothesis, we observed a ∼2-fold increase in the rate of IC-RP cleavage at 10 ng/ml TRAIL relative to 2 ng/ml and a further ∼1.5-fold increase at 250 ng/ml TRAIL (Figure 8B). To test directly the role of C8* in delay duration, we asked whether the short delay characteristic of cells exposed to 250 ng/ml TRAIL could be converted into a long delay by modulating C8* activity with a small-molecule inhibitor (Z-IETD-FMK; Figure 8C and 8D). At 10 μM, Z-IETD-FMK blocked C8 activation and MOMP in >95% of cells, as previously reported [59]. However, at 2 μM Z-IETD-FMK, C8* was only partially inhibited, and the rate of IC-RP processing in cells exposed to 250 ng/ml TRAIL was reduced to that of cells exposed to 50 ng/ml TRAIL. MOMP occurred under this condition, with T_d_ increased from 2.1 to 3.3 h (Figure 8D), thereby showing that dose-dependent changes in C8* activity are sufficient to alter T_d_.

### Prediction V: Ratiometric Control of Snap-Action Transmission

**Figure 9:**
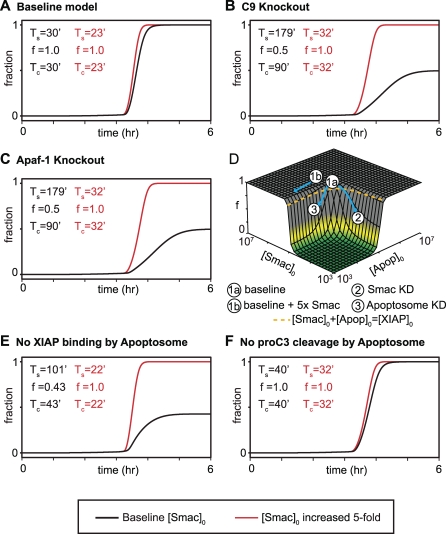
Role of the Apoptosome in Snap-Action C3 Activation (A–C) Simulation of the time course of C3 (EC) substrate cleavage in control cells (A), or cells lacking C9 (B), or Apaf-1 (C). For each condition, simulations were performed using baseline conditions for Smac (black lines), or 5-fold overexpression of Smac (red lines). (D) Simulation of *f* as a function of initial concentrations of Smac and the apoptosome; for simplicity, the apoptosome components C9 and Apaf-1 were assumed to be present at equal concentrations. Orange dotted line indicates the region where [XIAP]_0_ = [Smac]_0_ + [apoptosome]_0_; numbered circles and cyan arrows indicate the positions of the indicated conditions. Point 1a corresponds to the baseline model, in which either Smac or Apaf-1 knockdown leads to a decrease in *f*, while point 1b corresponds to the baseline model with 5-fold higher Smac, in which Apaf-1 depletion does not reduce *f*. (E, F) Simulation of the time course of PARP cleavage in cells in which apoptosome function has been altered so that it cannot process pro-C3 (E) or so that it cannot bind XIAP (F).

Two sets of reactions “transmit” the death signal generated by MOMP to C3*: (i) binding of cytosolic Smac to XIAP and (ii) assembly of the apoptosome. Smac binds and neutralizes XIAP, making it critical to C3* activation. The role of the apoptosome in extrinsic apoptosis is less clear, but simulation suggests that Apaf-1 and C9 should vary in importance depending on Smac levels. When Smac is present at high levels (5 × 10^5^ molecules/cell), removal of either Apaf-1 or C9 has little effect on *f*, T_s_, and T_d_ (32 min versus 23 min; Figure 9A–9C). However, at lower levels of Smac, depletion of apoptosome components abolishes snap-action C3* activation (*f* = 0.5, T_s_ > 100 min; in all cases, MOMP was unaffected by changes in Apaf-1/C9 levels). We hypothesize that the key quantity controlling signal transmission from MOMP to C3* is the ratio of XIAP to the sum of the Smac molecules and apoptosome complexes in the cytosol (Figure 9D, dotted line). In support of this idea, depletion of Apaf-1 by siRNA resulted in a loss of snap-action switching that could be reversed by simultaneous depletion of XIAP and intensified by simultaneous depletion of Smac (Figure S7). Unfortunately, we were not able to confirm this result with multiple Apaf-1 oligos and our prediction therefore remains only weakly substantiated. Nonetheless, model analysis shows that, under conditions of limiting Smac levels, the phenotype associated with a C9* variant unable to bind XIAP is similar to that of C9 or Apaf-1 deletion (Figure 9E), whereas C9* lacking catalytic activity does not substantially alter the kinetics of PARP cleavage (Figure 9F). Thus, we predict that the XIAP-binding properties of the apoptosome are more critical in extrinsic cell death than C3* activation and that other XIAP-binding proteins such as ARTS [60] and Omi/HtrA2 [61] may also be important. A proper exploration of “MOMP transmission” will therefore require measuring and manipulating the levels of multiple overlapping XIAP binding proteins.

### Prediction VI: C6*-Mediated Feedback Controls T_d_ but Not T_s_

**Figure 10:**
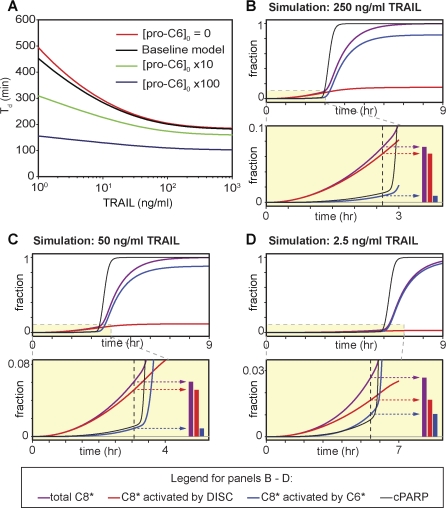
Role of C6 in Modulating the Duration of Pre-MOMP Delay (T_d_) (A) Simulation showing the effect on T_d_ of increasing the levels of [C6]_0_ 10- (green line) or 100-fold (blue lines) above baseline values (black line) or of eliminating C6 altogether (red line). (B–D) Simulations showing concentrations of total C8* (purple lines) and the subset of C8* generated by DISC (red lines) or by C6* (blue lines) at three different doses of TRAIL. The bottom panels magnify the period immediately before and after snap-action switching; bar plots to the right depict levels of DISC- or C6*-generated C8* (normalized to a total C8* value of 1.0) at the time of MOMP onset (dotted vertical lines).

Depletion of C6 10-fold by RNAi did not significantly alter the dynamics of effector caspase substrate cleavage in HeLa cells treated with TNF or TRAIL (Figure 5, relative to control depletions; T_d_ ≈ 80 min; T_s_ ≈ 15 min; *f* ≈ 1.0) and the lack of dependence on C6 was also captured by modeling (Figure 5). Remarkably, however, simulation showed C6* to play a major role in determining overall levels of C8* in dying cells. If the generation of C8* is separated into its two sources, DISC and C6*, it is apparent that a dramatic acceleration in C8* formation is induced upon C6* activation (Figure 10A). A priori, it might be assumed that this acceleration would constitute a key feature of snap-action switching. However, C8* generated by C6* lags temporally behind C8* generated by DISC and is present only after rapid cleavage of PARP has begun, making it inconsequential to the dynamics of TRAIL-mediated cell death under most circumstances (Figure 10B). As the dose of TRAIL is reduced, levels of DISC-generated C8* are also reduced, resulting in a larger relative contribution of C6* → C8 during the pre-MOMP period (Figure 10C and 10D). Thus, modeling predicts that C6 should have an impact on T_d_ at very low TRAIL concentrations. In addition, in cases in which [C6]_0_, or the rate of C8 cleavage by C6* is high, the dynamics of C3* substrate cleavage should depend on the C6* feedback loop. This prediction appears to be supported by data showing C6 overexpression to enhance the sensitivity of cells to various apoptotic stimuli [62]. We conclude that positive feedback mediated by C6 (or by other topologically analogous processes) plays a minor role in snap-action C3* activation in Hela cells but may be more important under other conditions, including type-I apoptosis, in which MOMP is not required [63].

### Building a Threshold-Sensitive Snap-Action Switch

**Figure 11:**
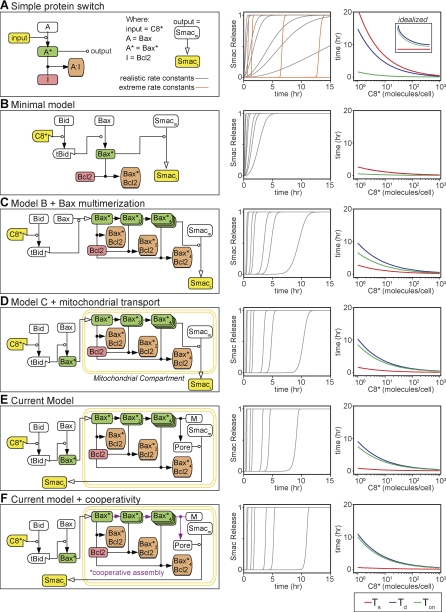
Role of Network Topology in Generating Snap-Action Behavior Models representing varying topologies of the MOMP module were analyzed for snap-action behavior. For each model, the input species (C8*) was introduced at values ranging from 1 to 10^3^ molecules/cell, and the resulting release behavior of Smac determined by simulation (middle column). The kinetic characteristics T_s_, T_d_, and T_on_ (defined as the time at which Smac release reached 1% of its final value) for Smac release were determined from these simulations as a function of input strength (right column). Note that *f* = 1 in all cases and therefore T_c_ = T_s_. Binding of Bid to Bcl-2 was omitted for simplicity, although this interaction is included in the full EARM v1.0. (A) Basic motif model. Simulations are shown for parameter sets representing physiologically realistic rate constants (gray) and for irreversible, faster-than-diffusion binding (orange) for Bax-Bcl-2 association, and T_s_, T_d_, and T_on_ curves shown only for physiologically realistic values. The inset plot in the right column shows the expected behavior for an idealized variable-delay snap-action switch. (B) Model of known topology, including Bid cleavage. (C) Model including Bax oligomerization. (D) Model including separate mitochondrial reaction compartment. (E) Model corresponding to the MOMP module in EARM v1.0. Note that the cytosolic pool of Bcl-2 anti-apoptotic proteins, which binds to tBid in EARM v1.0, has been omitted for simplicity. (F) Model as in (F), but with rate constants for Bax oligomerization and insertion adjusted to represent positive cooperativity.

Modeling strongly suggests that both the biochemistry and physical partitioning of apoptotic regulators play roles in MOMP. To undertsand these roles, we generated a series of models of increasing complexity that describe reactions linking C8* activation to cytosolic translocation of Smac/CyC; as described above, these are the reactions shown experimentally to transform a graded initiator caspase input into switch-like effector caspase output. The input-output responses of variant models were analyzed over a range of C8* input concentrations for T_d(MOMP)_, T_s(MOMP)_, and T_on(MOMP)_. The later metric represents the time at which 1% of Smac/CyC is translocated and is designed to capture the efficiency of repression in the pre-MOMP interval. In cells, T_s(MOMP)_ is short and invariant over a wide range of C8* levels (that is, “input-independence”), while T_d(MOMP)_ and T_on(MOMP)_ should be virtually identical and increase in a dose-dependent manner (Figure 11A, inset).

The simplest topology for a threshold-sensitive switch, and one implicit in the oft-cited concept of a Bax-Bcl-2 “rheostat” [64], involves an active species (Bax*) that is generated by an input (C8*) and then antagonized by binding to an inhibitor (Bcl-2). As Bax* increases in concentration, the pool of Bcl-2 is exhausted until [Bax*] > [Bcl-2] and the output (Smac*, representing cytosolic transport of Smac through Bax-containing pores) is induced. This system functions as a threshold-sensitive switch if Bax and Bcl-2 are assumed to associate at a rate faster than diffusion and to bind irreversibly (Figure 11A, orange curves). However, when realistic biochemical constants are used (e.g., a diffusion-limiting on-rate of ∼10^6^ M^−1^s^−1^ and K_d_s in the measured range of 1–10 nM, [7]), the circuit is ineffective as a switch (Figure 11A): T_s(MOMP)_ is highly input-dependent while T_on(MOMP)_ is always short. Addition of Bid-dependent Bax activation (C8* → tBid → Bax*; Figure 11B) and a further requirement for Bax multimerization during pore formation (Bax* → Bax_2_* → Bax_4_* → Smac*; Figure 11C) result in successive improvements in performance as judged by input-independence of T_s(MOMP)_ and input-dependence of T_on(MOMP)_. Further increases in the input-independence of T_s(MOMP)_ are achieved by introducing a mitochondrial membrane compartment in which Bax and Bcl-2 interact (Figure 11D). With Bax and Bcl-2 confined to a membrane compartment that is ∼7% of the cytosolic volume, protein concentrations and rates of association rise 14-fold. As a consequence, the Bax*-Bcl-2 association/dissociation reaction remains close to equilibrium even as free Bcl-2 falls to very low levels. A final improvement in performance is achieved if Bax_4_* is required to undergo a conformational change and membrane insertion reaction during pore formation (Figure 11E); motivation for this step comes from studies showing that Bax-mediated pores form only at specific sites in the mitochondrial outer membrane (an insertion step is likely to be even more importantfor Bak, which localizes to mitochondria even in normally growing cells) [65,66]. Overall, the steady improvement in performance observed with successive models is primarily attributable to imposition of tighter repression on pore formation during the delay phase (Figure S8); as noted above, very tight negative regulation of MOMP is critical to sustaining long T_d_.

Model E, which corresponds to the topology in EARM v1.0, generates T_d(MOMP)_, T_s(MOMP)_, and T_on(MOMP)_ that are largely consistent with the data, although T_s(MOMP)_ remains somewhat input-dependent at low TRAIL concentrations in the model but not in experiments. In model F, further input-independence in T_s(MOMP)_ can be achieved by inclusion of positive cooperativity in Bax oligomerization (this is modeled by making the rate constants of Bax dimerization, Bax tetramerization, and Bax insertion successively more rapid; Figure 11F). The impact of such cooperativity on the full ODE model remains to be determined, as does a demonstration that it exists in vivo. Nonetheless, it is noteworthy that robust all-or-none switching can be achieved in our simulations by a network that does not include feedback. It has been suggested that binding of Bcl-2 to both Bid and Bax multimers creates an “implicit positive feedback loop” [22,24], because the same pool of Bcl-2 inhibits sequential steps in the tBid → Bax* → Bax*_2_ → Bax*_4_ reaction series. However, implicit feedback does not appear to be essential for rapid switching, since model performance was not substantially impaired when separate, non-communicating, pools of Bcl-2 were implemented for all Bax-binding reactions (Figure S8). Instead, our findings suggest that snap-action control over MOMP arises from interplay between the biochemistry of protein-protein interaction and translocation between physical compartments of different volumes.

## Discussion

Variable-delay, snap-action regulation of effector caspases has been observed in a number of studies of extrinsic apoptosis, but the mechanisms responsible for this behavior have not, to our knowledge, been identified. Here, we develop and test an ODE-based model of pathways linking TRAIL-receptor binding to an extended pre-MOMP delay of variable duration followed by rapid snap-action cleavage of effector caspase substrates. Modeling cellular biochemistry inevitably involves a tradeoff between tractability, implying fewer species, and detail or scope, implying more species. Previously published models of caspase regulation by death receptors have focused on subsets of the reactions in EARM v1.0 including the C3/C6/C8 feedback loop [17], XIAP [22,23], C3 degradation [19], the apoptosome [20,67], or mitochondrial permeabilization [20,24–26]. However, our work suggests that models spanning reactions upstream and downstream of MOMP yield insight that cannot be obtained from models having less scope. In light of the the trade-off between scope and detail, it is interesting to note that significant mechanistic detail (e.g., multimerization, physical compartmentalization, etc.) was necessary to reproduce the features of TRAIL-induced MOMP identified experimentally. We therefore expect further insight as we add to EARM v1.0 details of DISC assembly [68], multiple initiator and effector caspases [3], and the 20 or so members of the Bcl-2-protein family [6]. Expanded models will be harder to manipulate and train but will make it possible to distinguish among alternate proposals for the reactions driving MOMP during intrinsic apoptosis and to explore the mechanisms of action of Bcl-2-binding drugs such as ABT-737 (see the EARM documentation page at http://www.cdpcenter.org for updates) [69].

Several hypotheses regarding the origins of snap-action control of apoptosis have emerged from previous studies, but relatively few have been explored experimentally at the single-cell level. Our primary guide has been single-cell data collected from normally growing and RNAi-depleted cells rather than mathematical analysis of EARM v1.0 equations (for which, after all, multiple formulations are possible). The importance of integrating modeling and experimentation is often emphasized [70] but is difficult to achieve in practice. One challenge is obtaining sufficiently accurate and quantitative experimental data, particularly for a process such as apoptosis that varies significantly from cell to cell. Here we use data fusion and simulation to integrate measurements made by live-cell imaging, flow cytometry, and immunoblotting with the result that mean values and variances could be obtained for key descriptors of C3* dynamics in normally growing and perturbed cells across a range of ligand concentrations. The use of synthetic data as a means to troubleshoot and analyze experiments is common in the measurement sciences, and the apparent effectiveness of this approach here and elsewhere [71] hints at its potential value in biology. A more fundamental challenge in linking simulations to experimental data is the difficulty of estimating unknown parameters. Given available data, EARM v1.0 is nonidentifiable and in the current work we rely on order-of-magnitude estimates. Singular perturbation analysis suggests that many model-based predictions are fairly robust to parametric uncertainty (J.M. Burke and P.K. Sorger, unpublished data), but further work is clearly required on this topic.

### Determinants of Snap-Action Activation

We find that MOMP is the point in the extrinsic apoptosis network at which a graded TRAIL → → C8* → → Bax* signal is transformed into an all-or-none snap-action signal. This manifests itself as sudden formation of GFP-Bax and GFP-Bak puncta, which are thought to correspond to clusters of pores [56], concomitant with rapid CyC and Smac translocation [47,48]. In contrast, cleavage of an IC-RP reporter (a proxy for the production of the Bax activator tBid) is gradual over many hours prior to MOMP. Our conclusion that snap-action behavior originates at the level of MOMP is consistent with previous single-cell [47,48,72] and biochemical [73,74] studies.

Reactions linking Smac and CyC translocation to effector caspase activation are also essential for execution of an all-or-none death decision. Data and modeling agree that depletion of Smac or overexpression of XIAP [23] creates a mismatch between XIAP levels and those of its binding partners, thereby preventing XIAP from being fully sequestered from C3* by proteins released during MOMP. Reactions involved in apoptosome assembly can potentially generate bistable or ultrasensitive behavior [20,22], but this capacity does not appear to be essential for snap-action C3 activation in our simulations: any such bistability manifests itself after the live-die decision is made.

To better understand how snap-action switching emerges from the reactions controlling MOMP, we have analyzed a series of models in which processes linking activation of C8* and release of Smac/CyC are represented with increasing complexity and realism. The models were scored for their ability to maintain a durable “off” state, a delay whose duration was determined by input strength (corresponding to TRAIL dose), and input-independence in the rate of transition from “off” to “on.” While a number of studies have characterized biochemical switches from the perspective of steady-state behavior and Hill functions [75,76], our metrics attempt to capture the temporal dynamics of switching, a topic that has received less attention [77]. An important but relatively obvious finding is that simple competition between an activator (Bax*) and an inhibitor (Bcl-2) makes for a poor switch under any set of realistic biophysical assumptions. Switch-like behavior relies on additional components, physical partitioning of reactants into compartments, and multimerization of Bax into functional complexes. One unusual aspect of MOMP is that its regulators are found in both the cytosol, where they diffuse in three dimensions, and the mitochdonrial membrane, where they diffuse in two dimensions. This transition could potentially restrict reaction volumes and further facilitate formation of the Bcl-2:Bax complexes critical for the inhibition of pore formation during the delay phase. When Bax- and Bak-containing pores finally form, rapid release of Smac/CyC is ensured by the favorable kinetics of moving concentrated protein stores down a steep concentration gradient (from the mitochondrial intermembrane space to the cytosol). Overshoot in the number of pores and rapid translocation kinetics do not guarantee complete input-independence of T_s(MOMP)_ in our models but do produce values in reasonable agreement with experimental data (5–10 min). However, at very low input levels, T_s(MOMP)_ in the model exceeds the range of values observed experimentally (increasing to ∼40 min). One potential way to overcome this is cooperativity in the Bax_1_* → Bax_n_* assembly reactions, but other mechanisms involving additional Bcl-2-like proteins also exist and require further exploration. Moreover, the remarkable overshoot in the number of pores formed relative to the number needed to translocate Smac/CyC raises the question of whether pores have another function or whether overshoot is an inevitable byproduct of rapid pore assembly. Finally, while the models described in this paper are effective at capturing the kinetics of apoptosis over relatively short time periods (6–24 h), protein synthesis and survival signaling must be added to capture long-term outcomes. Analysis of more complete models of MOMP, coupled with live-cell imaging of additional reactants, should help to further refine our understanding of how cells reconcile the competing demands of a long pre-MOMP delay and fast dose-independent switching.

### Feedback and Network Bistability

Biological switches involving changes in cell fate are often analyzed in terms of positive feedback and the potential for bistability [17,20,24]. However, we find that at least one large class of positive feedback mechanisms can be ruled out by our data: snap-action behavior at the level of MOMP occurs independently of caspase-dependent feedback. This finding, based on simultaneous monitoring of initiator caspases and MOMP in individual cells, is in agreement with data from others who have found that the kinetics of CyC release are unaffected by pan-caspase inhibition [48,72]. Moreover, we find no effect of depleting C6, a putative mediator of feedback from C3* to C8. These results do not rule out a role for local feedback in extrinsic cell death (involving, for example, the proposed conversion of Bax to Bax* by already active Bax* [24,58]). However, we find that even positive feedback loops that improve the dose-independence of T_s_ do so at the cost of reducing agreement between simulation and other experimental data, such as the dose-dependence of T_d_ and the kinetics of Bax cluster formation (unpublished data). Since putative switching mechanisms must be assessed in the context of all available data rather than their impact on T_s_ alone, our preliminary conclusion is that positive feedback in the induction of MOMP cannot be justified by existing data or simulations.

Bistability is an attractive concept in a network that decides between two fates, but does not appear to be important in our model. Protein synthesis and caspase-mediated feedback, necessary for bistability in many published models, are not required for snap-action behavior in our system. Moreover, even at the lowest TRAIL concentrations (in the presence of cycloheximide), all cells eventually die, implying the absence of a bifurcation in cell fate (this is not true for TNF, probably because of additional complexities in DISC formation). In agreement with this finding, singular perturbation analysis of EARM reveals the existence of a monostable transcritical bifurcation in the Bax-Bcl-2 binding reactions controlling MOMP rather than the bistable cusp bifurcation observed in pathways with strong feedback (J.M. Burke and P.K. Sorger, unpublished data). In rationalizing the absence of bistability, it is important to note that cell fate cannot be equated with the steady-state level of C3; under many conditions, transient effector caspase activation seals the fate of a cell long before steady-state is reached, with the result that a quasi-steady state having low C3* does not necessarily correspond to sustained survival [78]. It is clear however, that caspase networks play a role in cellular processes other than apoptosis [79,80], and it is possible that bistability arises in these networks in other physiological settings.

### Determinants of Variable Delay

What features of the pathways regulating extrinsic apoptosis control the pre-MOMP delay? One very stringent requirement is that no pores form during the delay phase since experiments and simulation suggest that a very small numbers of pores can kill a cell. During the delay, the C8* substrate tBid generates Bax*, which binds immediately to inhibitory Bcl-2 (in EARM v1.0 this takes place in the mitochondrial membrane compartment); when the active forms of Bax* exceed the number of Bcl-2, a threshold is reached, pores form, and cells proceed rapidly to death. We show by simulation and experiment that varying C8* activity is sufficient to explain the dose-dependence of T_d_. Model analysis indicates that the delay corresponds to the time necessary to generate enough Bax* to exhaust the pool of mitochondrial Bcl-2, in agreement with the long-standing notion that MOMP is controlled by the Bax:Bcl-2 ratio [81–83]. Although the available pool of mitochondrial Bcl-2 ultimately sets the threshold for pore formation, analysis predicts that changes in the levels of any protein upstream of Bax* can also affect delay. Increases in the levels of protective proteins such as FLIP, Bar, or cytosolic Bcl-2 should lengthen T_d_, while increases in pro-apoptotic proteins such as TRAIL receptor, C8, Bid, and Bax should shorten T_d_. Because caspase-mediated feedback can regulate delay at very low TRAIL concentrations (even if it does not play a role in snap-action switching), proteins downstream of MOMP such as C6, C3, and XIAP also have an impact on T_d_ under some circumstances. T_d_ also increases overall in cells that have not been treated with cycloheximide, largely as a consequence of a rise in the threshold at which MOMP occurs [15].

An oft-stated assumption about cellular networks is that they are robust to changes in protein concentrations and other parameter values [84]. Our results suggest that while T_s_ and *f* are robust to ligand dose and identity, T_d_ is sensitive to the concentrations of many reactants in the extrinsic cell death pathway and to external factors. The sensitivity of T_d_ can easily be rationalized as being important for a network that must adjust its responsiveness to different extra-cellular cues, depending on physiological context. Conversely, the robustness of T_s_ to natural variation in internal and external parameters is likely to be important in preventing accumulation of “half-dead” states. Ascertaining precisely how tumor cells exploit sensitive and robust features of cell death networks so as to escape normal control over proliferation promises to provide a new window into the role of apoptosis in oncogenic transformation.

### Physiological Importance of Variable-Delay Snap-Action Switching

At least three adaptive advantages might accrue to a cell in which apoptosis is under the control of a variable-delay, snap-action switch. First, the rapidity of switching should prevent cells from initiating but failing to complete the cell death program. Otherwise, a “half-dead” state might result, running the risk that chromosomal rearrangements and genomic instability typical of leukemia and other cancers might occur [14,85]. Second, dose-dependent variation in T_d_ should make it possible to regulate the strength of a death signal independent of an all-or-none response at the single-cell level. Longer delays prior to caspase activation are likely to correspond to lower overall sensitivity to cell death (under physiological conditions), since caspase activation pathways do not operate in isolation; induction of intracellular survival pathways can override TRAIL-induced death signals. Indeed, the number of cells committing apoptosis in response to death ligands can be modulated by treatment with EGF or insulin, which stimulate pro-survival pathways [86]. Because the duration of the pre-MOMP delay in TRAIL-treated cells is similar to the time required for induction of new protein synthesis, a race is run between pro- and anti-apoptotic processes, and longer delays in the pro-death cascade favor survival. Third, cell-to-cell variability in T_d_ within a population, a phenomenon observed even in the absence of new protein synthesis, prevents cells from dying en masse following exposure to a death stimulus*.* Recent work from our lab suggests that naturally occurring cell-to-cell variation in the concentrations of apoptotic regulators is responsible, in large part, for variability in T_d_ (S.L. Spencer and P.K. Sorger, unpublished data). We hypothesize that variability in T_d_ makes it possible for an all-or-none response at the single-cell level to be graded at the population level, such that the concentration of ligand controls the fraction of cells responding to a stimulus.

## Materials and Methods

### Reagents and cell culture.

HeLa cells stably expressing pMIG-Bcl-2 or pMIG (empty vector control) were gifts of Fei Hua and Michael Cardone; HeLa cells stably expressing EC-RP, IC-RP, and IMS-RP have been described elsewhere [15]. Plasmids containing the coding sequences of Bax and Bak were obtained from Richard Youle and the Harvard Institute of Proteomics. GFP-Bax and GFP-Bak were derived by PCR amplification of Bax and Bak coding sequences and ligation in frame with EGFP in pExchange-1 (Stratagene). HeLa cells stably expressing IMS-RP in combination with GFP-Bax and GFP-Bak were derived by standard transfection and selection techniques. Multiple stable clones were examined for each construct.

Antibodies were obtained as follows: cPARP, cleaved C3, XIAP, Bid, and Apaf-1 (BD Biosciences); C6, C8, and PARP (Cell Signaling); Smac (Calbiochem); Bax (Santa Cruz); cleaved cytokeratin (Roche). Secondary antibodies for flow cytometry and immunoblotting were from Molecular Probes (Invitrogen) and Rockland Immunochemicals. SuperKiller TRAIL was from Alexis Biochemicals, and cycloheximide (CHX) was from Sigma. siRNA oligos were purchased from Dharmacon; sequences are listed in Table S7.

### Live cell microscopy.

HeLa cells stably expressing fluorescent reporters were grown in chambered coverglass slides (LabTek) for 24–48 h. Immediately prior to imaging, medium was replaced with phenol red-free CO_2_-independent medium (Gibco) containing the appropriate concentrations of SuperKiller TRAIL and CHX. Cells were then imaged at 37 °C on a Deltavision Spectris AX71 microscope equipped with a temperature control chamber using 10×, 20×, or 60× objectives. CFP, YFP, GFP, or RFP images were acquired at 3-min intervals for 12–18 h. Cleavage of the reporters, which results in a separation of the CFP and YFP proteins and loss of intramolecular FRET, was monitored by the change in CFP:YFP ratio. The changes in CFP:YFP ratio over time for individual cells were measured using ImageJ software with a custom-made plugin, which is available from the authors, and T_d_ and T_s_ were determined by fitting cells to Equation (1) in Matlab (for T_s_, time courses were fit using only data prior to cell shrinkage, since data after this point were unreliable). Mitochondrial localization of IMS-RP, GFP-Bax, and GFP-Bak was assessed by quantitating the total intensity for individual cells after images were processed using the “Find Edges” algorithm in ImageJ. Due to photobleaching, analysis of the IMS-RP signal in each cell was limited to the period beginning 1–2 h prior to MOMP and ending with the appearance of apoptotic morphology; no significant changes in IMS-RP localization were visible outside of this period. Rate of release of IMS-RP was computed as the difference in the edge-detected signal for successive frames. For alignment of multiple cells, T_d(MOMP)_ for each single cell time course was set to the population average value for T_d(MOMP)_, and the average FRET cleavage ratio was then calculated for each time point.

### siRNA transfection.

siRNA transfections were performed as described [87], with a final concentration of 3–5 nM in the growth medium for each oligo. Medium was changed one day after transfection, and TRAIL treatment was performed on the second day after transfection. Oligo sequences are shown in Table S7.

### Immunoblotting.

Immunoblots were carried out as described [88], detected using an Odyssey Scanner (Li-Cor Instruments), and quantitated using ImageJ.

### Inclusion of cycloheximide in death assays.

All TRAIL or TNF treatments were carried out with 2.5 μg/ml CHX present to inhibit protein synthesis. Cells treated with 2.5 μg/ml CHX alone showed no evidence of PARP cleavage or cell death for at least 24 h.

### Flow cytometry assays.

Fixation, staining, and analysis procedures for flow cytometry were performed as previously described [15]. Antibody concentrations were 1:250 for anti-cleaved PARP and 1:500 for anti-cleaved C3 and anti-cleaved cytokeratin. Analysis was performed using the FlowJo software package (Treestar).

### Simulation of flow cytometry data.

Simulated live-cell, flow cytometry, and immunoblot data were produced using the Matlab program simFACS, which is included as supplementary Protocol S2. Estimation of T_d_, T_s_, and *f* was performed by manually adjusting these parameters to fit experimentally observed frequencies of negative, intermediate, and positive cells.

### Mathematical model.

Biochemical reaction equations were derived from the canonical death receptor network (see [89] for review). For each given binary reaction *i* the biochemical equation is represented by one of following general mass-action paradigms:


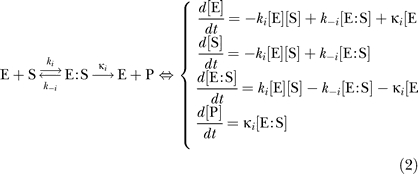



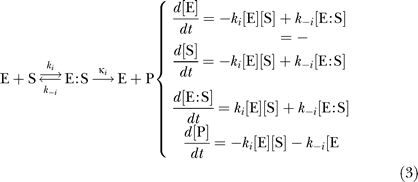



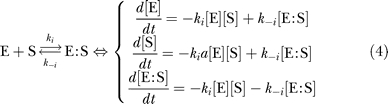


where an enzyme or other protein *E* reacts with its substrate or binding partner *S* forming complex *E:S*, and depending on the specific reaction, *i* forming product *P* such that *k*_i_, *k*_−i_, and κ_i_ are the forward, backward, and catalytic rates, respectively. Tables S1 and S2 list the species that are used in the biochemical equations.

Cytosolic and mitochondrial compartments (see below) are each assumed to be well mixed. Let *x* = [*X*] represent the number of molecules of species *X* in each compartment. Let the rate at which the number of molecules per compartment of species *X* changes with respect to time *t*, in seconds, be represented by *x* = *dx/dt*. Using the rates found in Table S3, and the state variables and initial conditions in Table S5, the biochemical reactions in Table S2 are modeled by the system of ODEs found in Table S6.

### Simulation and visualization.

Matlab stiff solver ODE15s was used to numerically solve the system of ODEs; the full model and Matlab routines for producing all simulations are contained in Protocol S1. Graphs and visualizations were made using Matlab and Axum software packages. Experimental overexpression and knockdown perturbations are simulated in the model by adjusting the baseline initial conditions appropriately in accordance with immunoblot data (Figure S2).

### Cytosolic and mitochondrial compartments.

Since many of the biochemical processes of the mitochondrial pathway occur on or in the outer membrane of the mitochondria, these processes are effectively physically separated from processes occurring in the cytosol. To reflect this segregation, the model is comprised of two compartments, cytosolic and mitochondrial. Each compartment is a separate, well-mixed volume; specific molecules, such as CyC, may move from one compartment to another at a defined rate. Based on measurements of the mitochondrial and cytosolic volume of HeLa cells [90], the volume of the mitochondrial compartment is estimated to be ∼7% of the volume of the cytosolic compartment. The rates of second-order (or higher-order) reactions, in which two molecules must collide, increase as volume decreases, due to the increased likelihood of a collision. To reflect this, the reaction rates of intermolecular reactions occurring in the mitochondrial compartment are divided by the ratio of the mitochondrial compartment volume to the cytosolic compartment volume (*v* = 0.07).

### Rate constants.

We assume a cellular volume of 10^−12^ liters (1 pL), and diffusion-limited bimolecular reaction rate constants *k*_i_. Therefore the forward reactions are bound by *k*_i_ ≤ 4.2 × 10^6^ M^−1^ s^−1^ and 4.2 × 10^4^ M^−1^ s^−1^ in the cytosolic and mitochondrial compartments, respectively, for all reactions *i* [91,92]. All dissociation rates are approximately 10^−3^ s^−1^, resulting in dissociation constants in the range of 1–10 nM in the cytosolic compartment (with one exception: for activation of receptor the dissociation rate is 10^−5^ s^−1^, since this is a lumped parameter fit to match data). All catalytic rates are 0, 1, 0.1, or 10 s^−1^. All translocation reactions (between cytosol and mitochondrial compartments) have a rate of 10^−2^ s^−1^.

### Initial protein concentrations.

Relatively few references for initial concentrations of proteins are available (Table S5; note also that values used in the model do not always correspond to those in the provided references due to fitting). However, many signaling proteins have been found to be present in the range of 1nM to 1μM (∼10^3^–10^6^ copies per cell) [93]. Therefore, we restricted initial protein concentrations to fall within these bounds (with the exception of c-Flip, which does not figure significantly in the model and which was estimated to be present at lower levels).

### Protein synthesis.

To reflect the use of cycloheximide in all experiments, the mathematical model contains no source terms.

### Protein degradation.

Protein degradation is assumed to be slow compared to signaling dynamics, so with one exception, the model contains no sink terms. The one exception is the case of C3* degradation by XIAP, which was experimentally determined to occur at significant levels on the timescale of the experiment [15]. In this case, degradation is approximated by assuming that ubiquitinated C3 is catalytically inactive.

### MOMP variant models.

Models were constructed corresponding to the diagrams shown in Figure 11. Rate constants and initial conditions for Bid, Bax, Bcl-2, and mitochondrial binding sites were identical to the corresponding parameters in EARM v1.0 unless otherwise indicated. For simplicity, MOMP was represented by the release of Smac alone, with an initial concentration of 10^6^ molecules/cell.

## Supporting Information

Figure S1 Observation of Snap-Action Behavior in Multiple Endogenous Effector Caspase Substrates HeLa cells were treated with 50 ng/ml TRAIL + 2.5 μg/ml CHX for the indicated times, and then analyzed by flow cytometry for anti-cleaved PARP or anti-cleaved cytokeratin antibodies. The data were first gated to exclude debris with low FSC signal (top panels, blue boxes) and then plotted as histograms (lower panels). In the case of both cleaved PARP and cleaved cytokeratin, >95% of cells display either positive or negative staining at all times, with <5% of cells falling into the intermediate range. (Note however, that the position and width of the positive population is different for the two antigens, probably as a result of differences in antibody affinity and subcellular distribution of the antigens.)
(296 KB AI)

Figure S2 Measurement of Perturbation Magnitudes (A) Quantitation of protein depletion and overexpression by immunoblot. For each siRNA oligo used, HeLa cells were transfected as described in the Methods section. After ∼48 h, cells were lysed in sample buffer and separated by SDS-PAGE. Blots were then probed with an antibody against the targeted protein (top image in each panel) and an antibody against a nontargeted protein as a loading control (bottom image in each panel). The level of depletion induced by each oligo (relative to nontargeting control oligo, and corrected for loading where possible) was quantitated using ImageJ software and is shown beneath each lane.
(B,C) Assessment of knockdown homogeneity by flow cytometry. HeLa cells were transfected with XIAP siRNA as in (A) and then trypsinized, fixed, and analyzed for XIAP levels by flow cytometry. Data are shown either as histograms (with data collected in linear amplification mode [B]), or scatter plots (with data collected in logarithmic amplification mode [C]; blue region denotes the untransfected population). Analysis of the fraction of untransfected cells (percentages listed above gated regions) indicated that knockdown occurred in 90%–95% of cells under typical conditions.
(394 KB AI)

Figure S3 Agreement between Simulated and Experimental Flow Cytometry Data Complete timecourses for each of the perturbations shown in Figure 5; simulated distributions were computed using simulated values for T_s_, T_d_, and *f* (from EARM v1.0) as input into the flow cytometry data simulation (see Protocol S2). Although the overall qualitative agreement between simulation and experiment is high, several points of discrepancy can be observed. First, the cell-to-cell variability parameters assumed by the flow cytometry simulation are not appropriate under all perturbed conditions. For example, under Smac depletion conditions, the assumed variance in T_d_ is too great at early times, causing a shift in the unstimulated (0 h) population that is not observed experimentally, while the assumed variance in *f* at late times is too small, leading to an equilibrium population (4.5 h) that is narrower than observed experimentally. Furthermore, while measurement noise is assumed to be the same for all cells in the flow cytometry simulation, experimental data suggest that there is considerably more variation in the measured values for cPARP-positive cells than for cPARP-negative cells (compare negative and positive peaks for control cells), which is likely explained by the increased morphological heterogeneity of cells that are being actively degraded in the later stages of apoptosis. Second, the shift in cPARP fluorescence in MOMP-blocked cells (Bid-depleted or Bcl-2-overexpressing) is larger in EARM v1.0 than seen in experiment; this shift is due to the difficulty of maintaining an effective C3* “off” state using reported values for cellular XIAP and C3 concentrations, which we have discussed in detail elsewhere [15]. Third, in Bcl-2-overexpressing, XIAP-depleted cells, model and experiment display minor differences in *f* (0.32 versus 0.15 for 87% XIAP depletion, and 0.57 versus 0.3 for 94% depletion) that appear exaggerated on a log scale, despite the fact that the model accurately captures the key qualitative behavior of the system, switching with *f* < 1. Thus, the model is in reasonable quantitative agreement with experimental observations, but additional calibration of both EARM v1.0 and the data simulation model may eventually yield closer matches.
(1 MB AI)

Figure S4 Multiple-Species Simulation in the Presence of Feedback The simulations shown in Figure 6A were also run in the presence of C6-mediated feedback. (Feedback was omitted in Figure 6A to make clear that the observed kinetics were feedback-independent). Very similar kinetics are observed whether C6-mediated feedback is present or absent. The largest differences are seen in the kinetics of C8* and tBid, which show a second, rapid phase of activation following MOMP in the presence of feedback.
(302 KB AI)

Figure S5 Dependence of Smac Release Kinetics on the Size of the Mitochondrial Smac Pool Simulations were performed under identical conditions to those in Figure 6D, except that the initial concentration of Smac in the mitochondria was increased to 10^9^ molecules/cell and CyC was set to 0 (so that the total pool of molecules to be released was 10^9^). Under these conditions, Smac release takes much longer (∼2 h, compare to Figure 6D), and many more pores are involved in the release event. This result confirms that the rapid, switch-like kinetics of Smac release seen with baseline Smac concentration (Figure 6D) require that the capacity of mitochondrial pores be in large excess relative to the size of the Smac pool to be released.
(234 KB AI)

Figure S6 Formation of GFP-Bak Puncta In Synchrony with MOMP HeLa cells expressing GFP-Bak and IMS-RP were treated with TRAIL and imaged at 30-s intervals with a 60× objective. The localization of GFP-Bak and IMS-RP are shown as raw images and as processed images after the application of an edge-detection filter. The first frame in which IMS-RP is detected is highlighted by a yellow box; in the same frame, the first GFP-Bak puncta appear (arrow heads). Thus, as expected for functionally redundant proteins involved in pore formation, GFP-Bax and GFP-Bak both form oligomeric clusters simultaneous with the initiation of MOMP and continue to form clusters long after MOMP has been completed.
(4 MB AI)

Figure S7 Effects of Apaf-1 Depletion (A–C) HeLa cells were transfected with siRNA oligos targeting Apaf-1 (A), Apaf-1 and XIAP (B), or Apaf-1 and Smac (C), treated with 50 ng/ml TRAIL, and assayed for cPARP by flow cytometry.
(D) Quantitation of Apaf-1 knockdown by immunoblot. For one Apaf-1 oligo (oligo number 1 in [D]), Apaf-1 produces similar results to Smac depletion (Figure 5), resulting in a persistent population of intermediate-cPARP cells that indicates a decrease in *f* (A). Co-depletion of XIAP and Apaf-1 restores efficient PARP cleavage (B), while codepletion of Smac and Apaf-1 has an additive inhibitory effect on PARP cleavage (more cPARP-negative cells persist at longer times [C]). These results favor a model in which both Smac and the apoptosome cooperate to antagonize XIAP, so that depletion of either Smac or the apoptosome component Apaf-1 results in a decrease in *f* (point 1 in Figure 9D). However, other Apaf-1 oligos, which appeared to deplete Apaf-1 with similar efficiency (D), did not produce a significant change in *f*, and did not act in an additive manner with Smac codepletion (not shown). Thus, the role of the apoptosome in snap-action switching cannot be conclusively determined from these results. Nonetheless, EARM v1.0 can be reconciled with both a scenario where the apoptosome plays a significant role, and one in which it does not (Figure 9D).
(852 KB AI)

Figure S8 Role of Bcl-2/Bax Configuration in Maintaining the Off-State of the Snap-Action Switch Using the current MOMP module (Model E in Figure 11) as a starting point, several variants were examined.
(A) Inhibition of Bax oligomeric forms by distinct pools of Bcl-2. Although it is more likely that a common pool of anti-apoptotic Bcl-2-like proteins bind to all oligomeric forms of Bax, we examined the behavior of a model in which a separate pool of Bcl-2 proteins mediates inhibition of each successive oligomeric form of Bax. Although this model is less plausible, it removes the potential for “implicit feedback,” in which upstream steps of Bax oligomerization are accelerated by the sequestration of Bcl-2 by downstream oligomeric forms. Switching behavior is only slightly impaired in this model, indicating that implicit feedback is not a central component of the switching seen in Model E. Moreover, the slight loss of snap-action behavior is attributable, at least in part, to factors other than implicit feedback, such as the fact that the separate pools of Bcl-2 do not reach the point of depletion simultaneously.
(B) Effect of selective inhibition of Bax oligomeric forms. The six models shown are identical to that in the top panel of (A), with the exception that Bcl-2 was only permitted to bind to the oligomeric forms of Bax shown in the corresponding diagrams. In nearly all cases, snap-action behavior is impaired relative to a model in which Bcl-2 binds to all oligomeric forms of Bax (A, top), with models in which Bcl-2 inhibits only a single oligomeric form of Bax* performing weakest. Therefore, the ability of Bcl-2 to bind to multiple oligomeric forms of Bax may contribute substantially to snap-action behavior.
(663 KB AI)

Protocol S1 A Matlab File Containing the Full EARM v1.0 Model and Routines for Producing the Simulations Shown in Figures 4–10
Instructions for choosing which simulations to run are included within the file.
(44 KB TXT)

Protocol S2 A Matlab Program, simFACS, for Producing Simulated Single Cell, Flow Cytometry, and Immunoblot Data Running the program produces a graphical interface with user-editable fields for adjusting all switching parameters; pressing the “Plot” button updates the visualizations.
(63 KB TXT)

Table S1 List of Species in EARM v1.0 (67 KB PDF)

Table S2 EARM v1.0 Biochemical Reactions (86 KB PDF)

Table S3 EARM v1.0 Rate Constant Values (75 KB PDF)

Table S4 References for Rate Constants (374 KB PDF)

Table S5 Variable Definitions and Initial Concentrations for EARM v1.0 (333 KB PDF)

Table S6 EARM v1.0 Equations (68 KB PDF)

Table S7 Sequences of siRNA Oligonucleotides (16 KB PDF)

## Abbreviations

C3: caspase-3
C6: caspase-6
C7: caspase-7
C8: caspase-8
C9: caspase-9
CyC: cytochrome c
DISC: death-inducing signaling complex
EARM: extrinsic apoptosis reaction model
EC-RP: effector caspase reporter protein
FRET: Förster resonance energy transfer
GFP: green fluorescent protein
IAP: inhibitor of apoptosis protein
IC-RP: initiator caspase reporter protein
IMS-RP: mitochondrial intermembrane space reporter protein
MOMP: mitochondrial outer membrane permeabilization
ODE: ordinary differential equation
RNAi: RNA interference
siRNA: small interfering RNA
TNF: tumor necrosis factor
TRAIL: TNF-related apoptosis-inducing ligand
