# Supplementary material for: Modeling a Snap-Action, Variable-Delay Switch Controlling Extrinsic Cell Death
Source: PLoS Biol. 2008 Dec 2;6(12):e299. doi: 10.1371/journal.pbio.0060299 (PMC2592357; doi:10.1371/journal.pbio.0060299)
Supplement: Supplementary file 1 [file pbio.0060299.sg001.pdf]

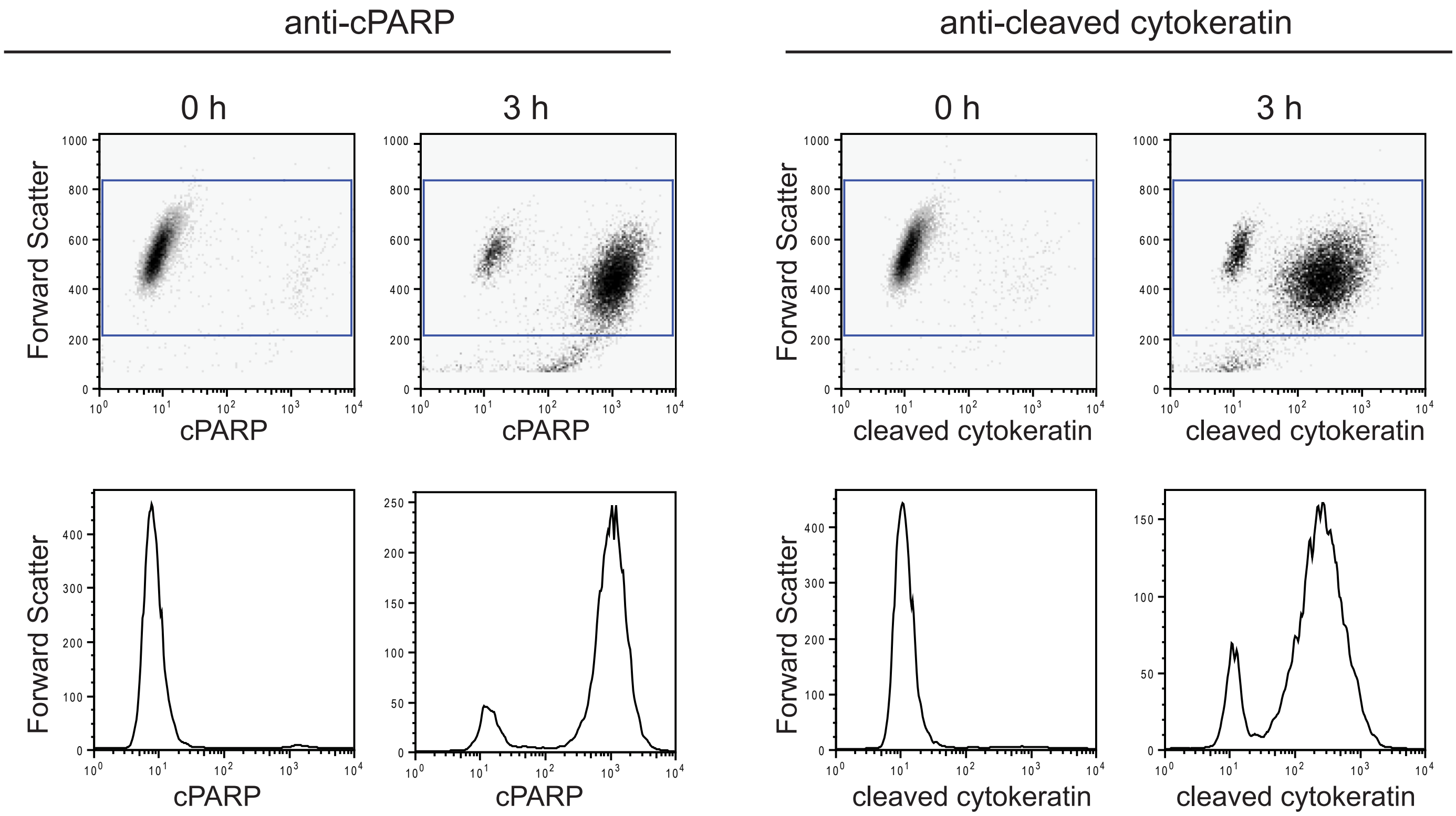
anti-cPARP
anti-cleaved cytokeratin
0 h
3 h
0 h
3 h
Forward Scatter
cPARP
cleaved cytokeratin
