# Supplementary material for: Modeling a Snap-Action, Variable-Delay Switch Controlling Extrinsic Cell Death
Source: PLoS Biol. 2008 Dec 2;6(12):e299. doi: 10.1371/journal.pbio.0060299 (PMC2592357; doi:10.1371/journal.pbio.0060299)
Supplement: Supplementary file 2 [file pbio.0060299.sg002.pdf]

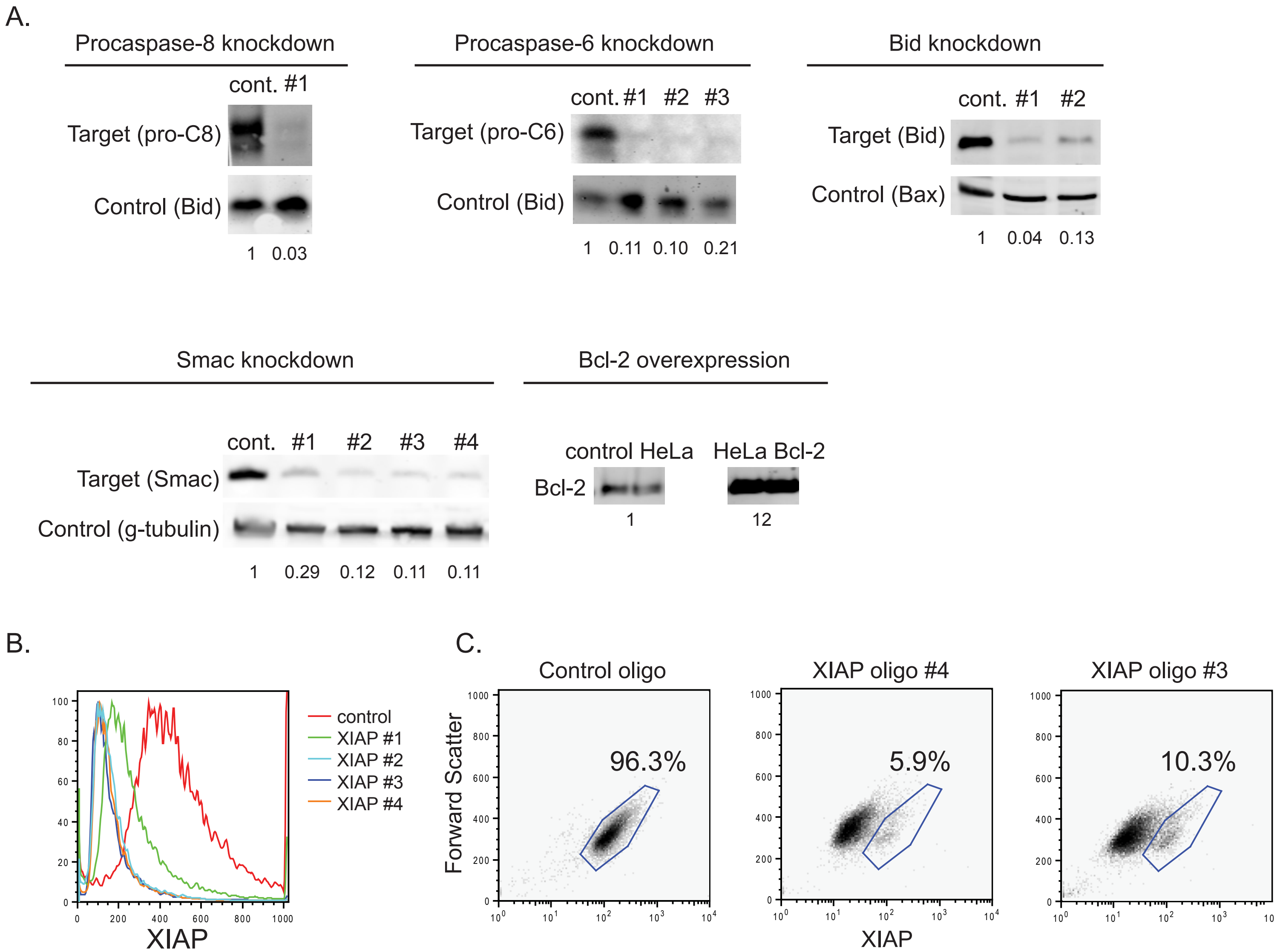
A.
Procaspase-8 knockdown
cont. #1
Target (pro-C8)
Control (Bid)
1 0.03
Procaspase-6 knockdown
cont. #1 #2 #3
Target (pro-C6)
Control (Bid)
1 0.11 0.10 0.21
Bid knockdown
cont. #1 #2
Target (Bid)
Control (Bax)
1 0.04 0.13
Smac knockdown
cont. #1 #2 #3 #4
Target (Smac)
Control (g-tubulin)
1 0.29 0.12 0.11 0.11
Bcl-2 overexpression
control HeLa HeLa Bcl-2
Bcl-2
1 12
B.
control
XIAP #1
XIAP #2
XIAP #3
XIAP #4
XIAP
C.
Control oligo
XIAP oligo #4
XIAP oligo #3
Forward Scatter
96.3%
5.9%
10.3%
XIAP
