# Supplementary material for: Modeling a Snap-Action, Variable-Delay Switch Controlling Extrinsic Cell Death
Source: PLoS Biol. 2008 Dec 2;6(12):e299. doi: 10.1371/journal.pbio.0060299 (PMC2592357; doi:10.1371/journal.pbio.0060299)
Supplement: Supplementary file 3 [file pbio.0060299.sg003.pdf]

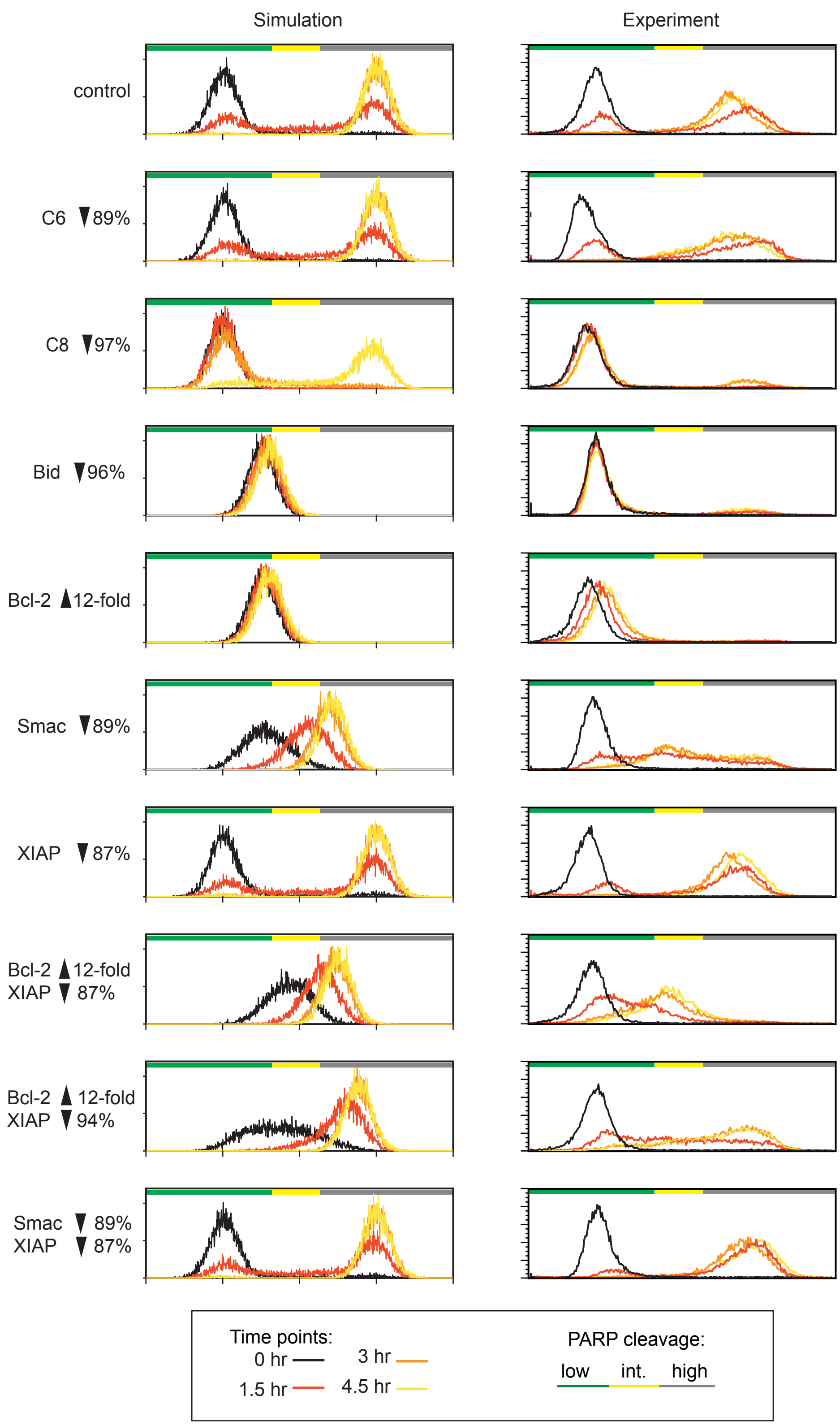
Simulation
Experiment
control
C6 ▼89%
C8 ▼97%
Bid ▼96%
Bcl-2 ▲12-fold
Smac ▼89%
XIAP ▼87%
Bcl-2 ▲12-fold
XIAP ▼ 87%
Bcl-2 ▲ 12-fold
XIAP ▼ 94%
Smac ▼ 89%
XIAP ▼ 87%
Time points:
0 hr
3 hr
1.5 hr
4.5 hr
PARP cleavage:
low
int.
high
