# Supplementary material for: Modeling a Snap-Action, Variable-Delay Switch Controlling Extrinsic Cell Death
Source: PLoS Biol. 2008 Dec 2;6(12):e299. doi: 10.1371/journal.pbio.0060299 (PMC2592357; doi:10.1371/journal.pbio.0060299)
Supplement: Supplementary file 4 [file pbio.0060299.sg004.pdf]

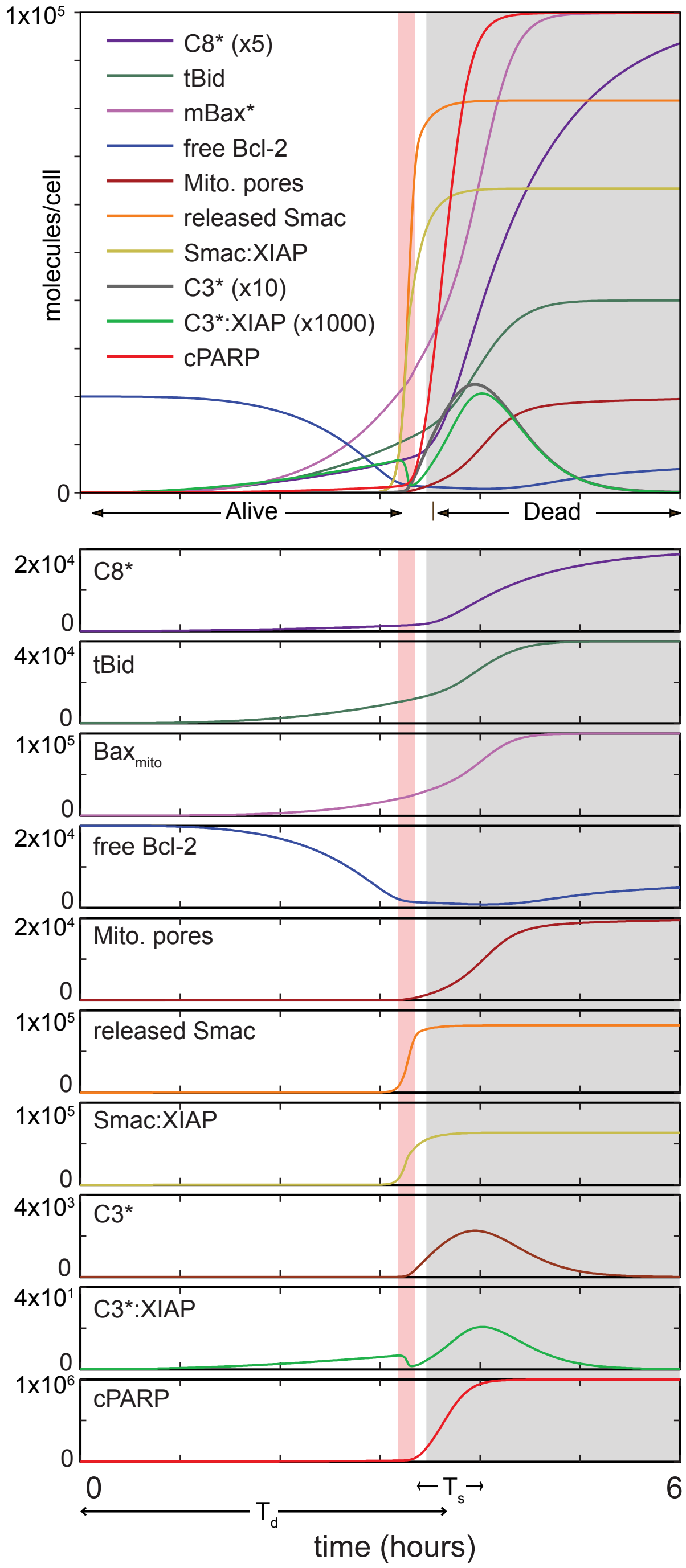

1x10^5
C8* (x5)
tBid
mBax*
free Bcl-2
Mito. pores
released Smac
Smac:XIAP
C3* (x10)
C3*:XIAP (x1000)
cPARP
molecules/cell
0
Alive
Dead
2x10^4
C8*
0
4x10^4
tBid
0
1x10^5
Bax_mito
0
2x10^4
free Bcl-2
0
2x10^4
Mito. pores
0
1x10^5
released Smac
0
1x10^5
Smac:XIAP
0
4x10^3
C3*
0
4x10^1
C3*:XIAP
0
1x10^6
cPARP
0
0
T_s
6
T_d
time (hours)
