# Supplementary material for: Modeling a Snap-Action, Variable-Delay Switch Controlling Extrinsic Cell Death
Source: PLoS Biol. 2008 Dec 2;6(12):e299. doi: 10.1371/journal.pbio.0060299 (PMC2592357; doi:10.1371/journal.pbio.0060299)
Supplement: Supplementary file 5 [file pbio.0060299.sg005.pdf]

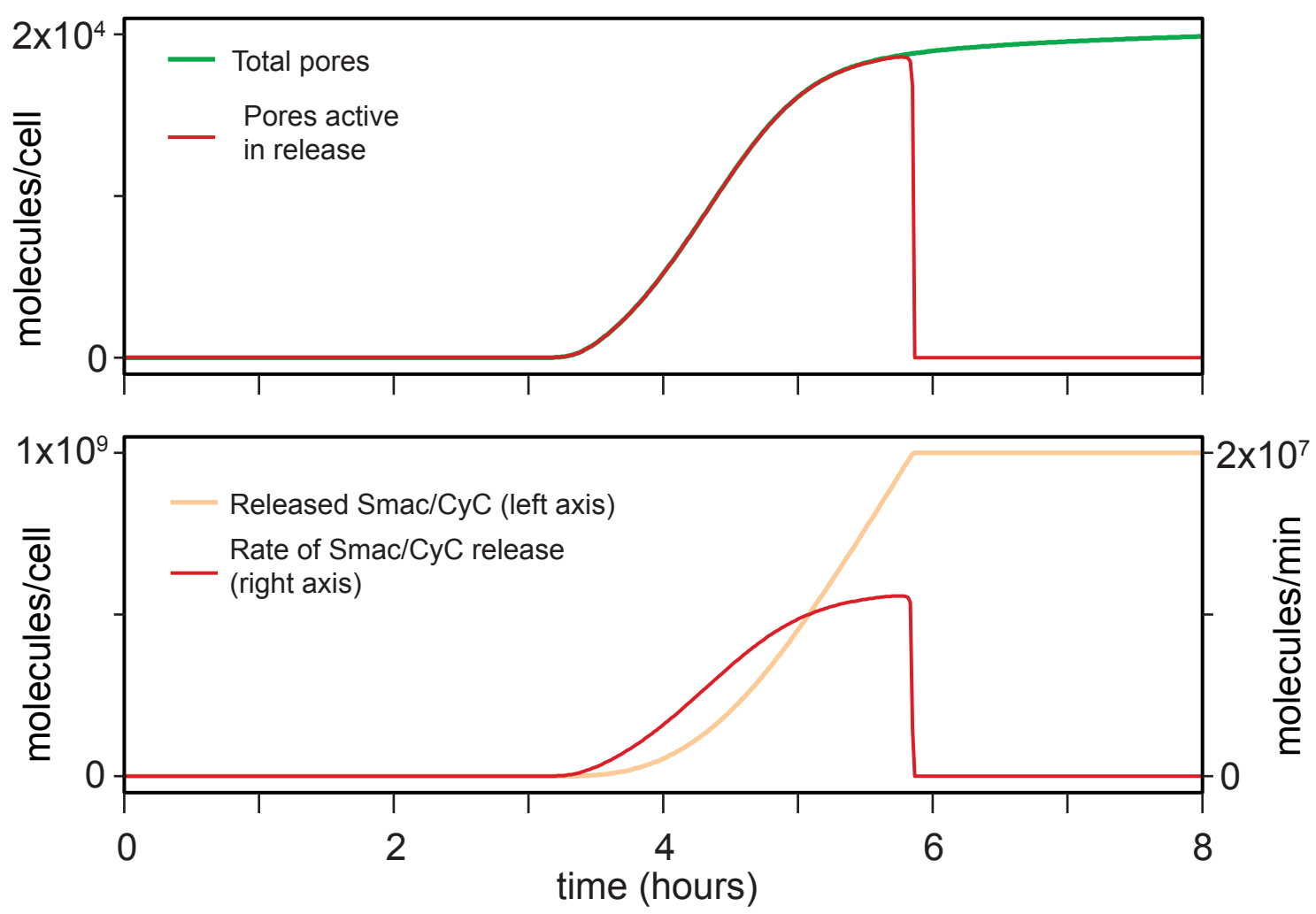

2x10^4
0
molecules/cell
Total pores
Pores active
in release
1x10^9
0
molecules/cell
2x10^7
0
molecules/min
Released Smac/CyC (left axis)
Rate of Smac/CyC release
(right axis)
0
2
4
6
8
time (hours)
