# Supplementary material for: Modeling a Snap-Action, Variable-Delay Switch Controlling Extrinsic Cell Death
Source: PLoS Biol. 2008 Dec 2;6(12):e299. doi: 10.1371/journal.pbio.0060299 (PMC2592357; doi:10.1371/journal.pbio.0060299)
Supplement: Supplementary file 6 [file pbio.0060299.sg006.pdf]

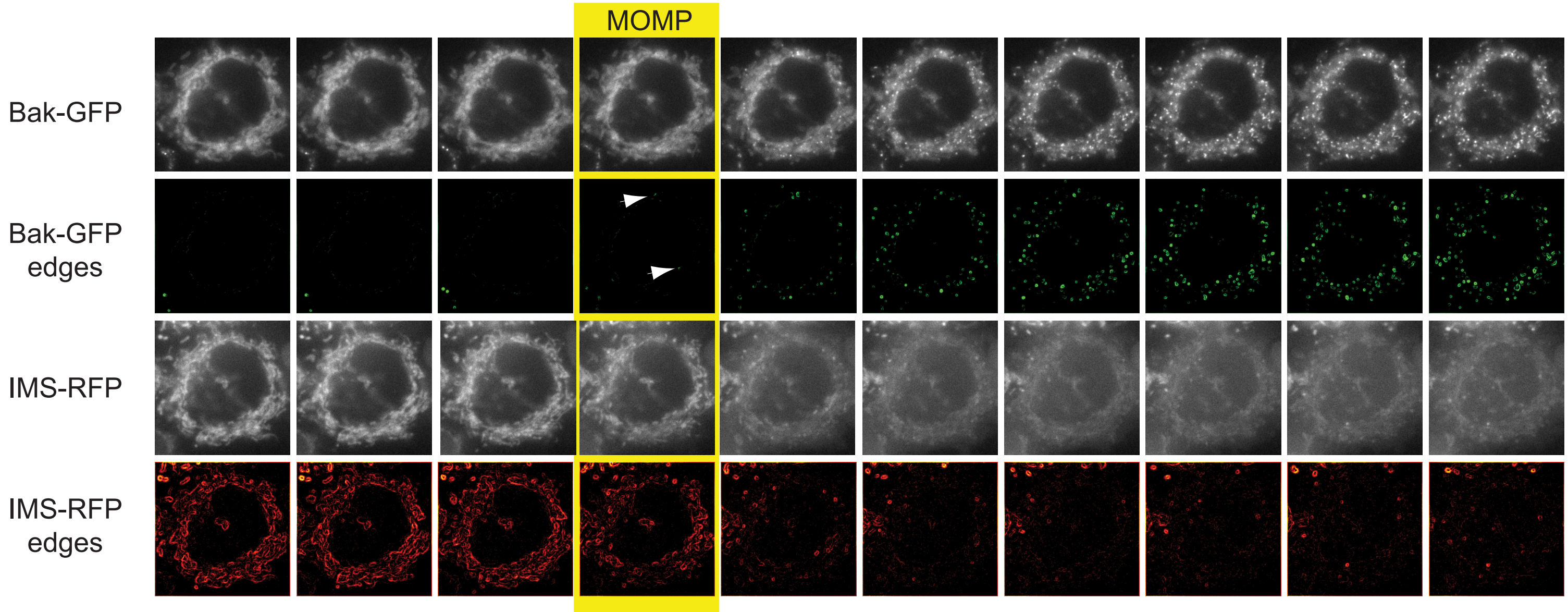
MOMP
Bak-GFP
Bak-GFP edges
IMS-RFP
IMS-RFP edges
