# Supplementary material for: Modeling a Snap-Action, Variable-Delay Switch Controlling Extrinsic Cell Death
Source: PLoS Biol. 2008 Dec 2;6(12):e299. doi: 10.1371/journal.pbio.0060299 (PMC2592357; doi:10.1371/journal.pbio.0060299)
Supplement: Supplementary file 7 [file pbio.0060299.sg007.pdf]

A.

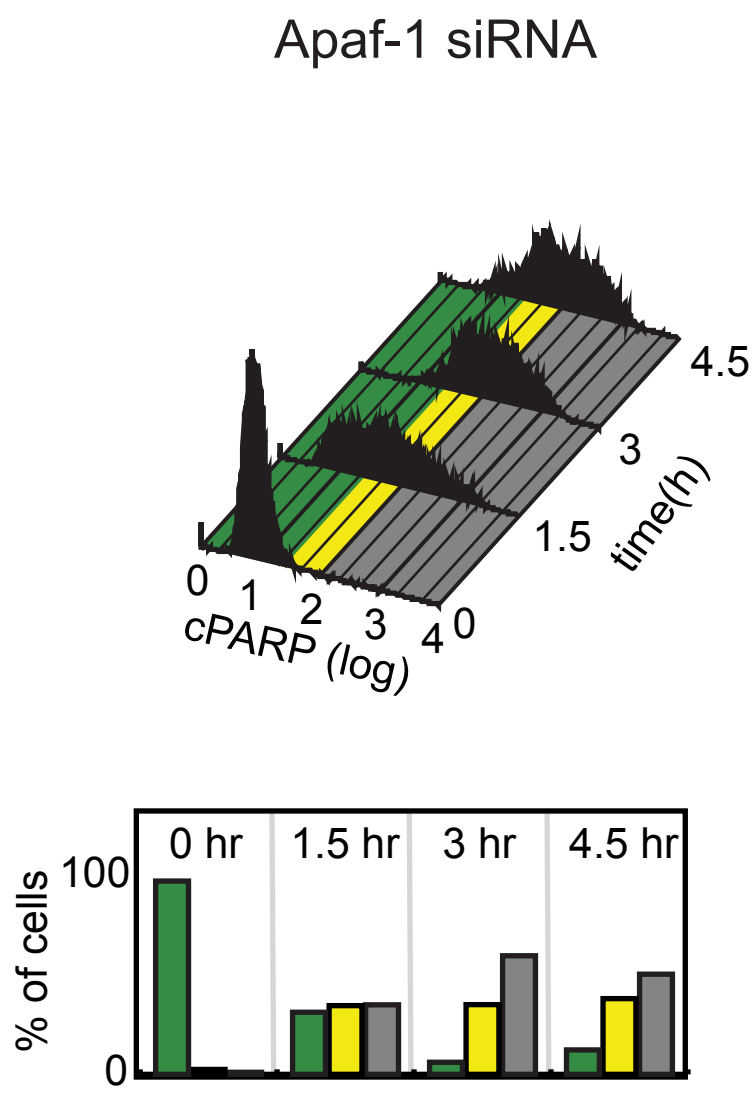
Apaf-1 siRNA
0 1 2 3 4
cPARP (log)
0 1.5 3 4.5
time(h)
% of cells
100
0
0 hr
1.5 hr
3 hr
4.5 hr

B.

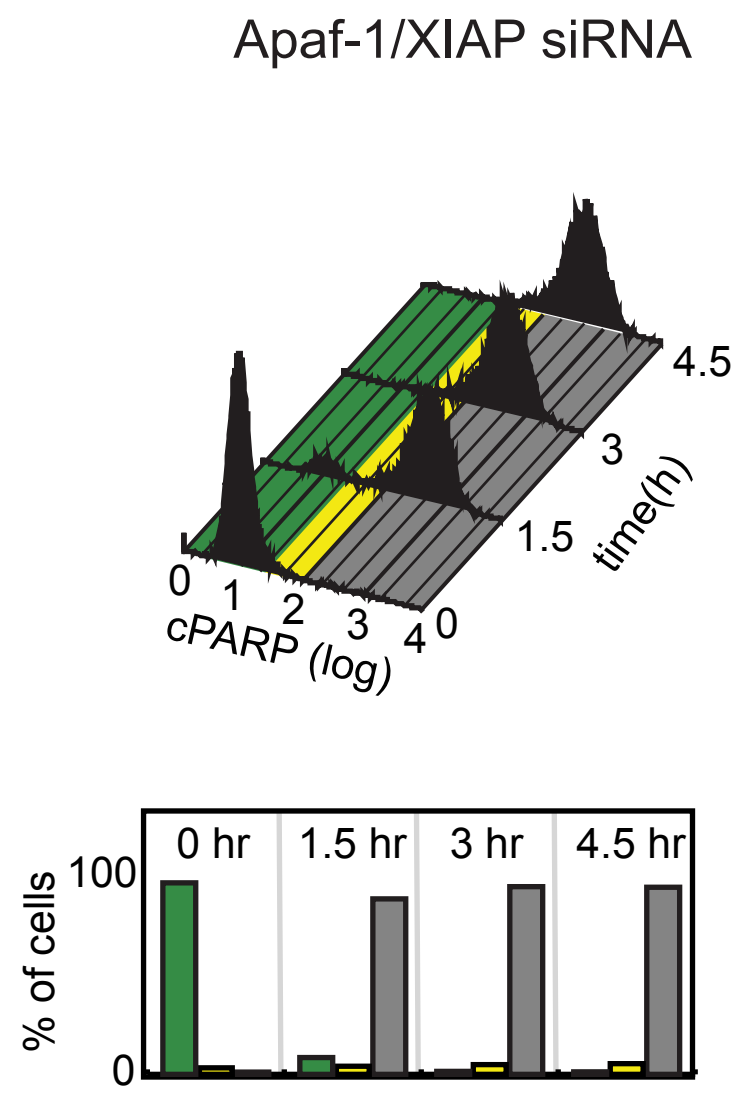
Apaf-1/XIAP siRNA
0 1 2 3 4
cPARP (log)
0 1.5 3 4.5
time(h)
% of cells
100
0
0 hr
1.5 hr
3 hr
4.5 hr

C.

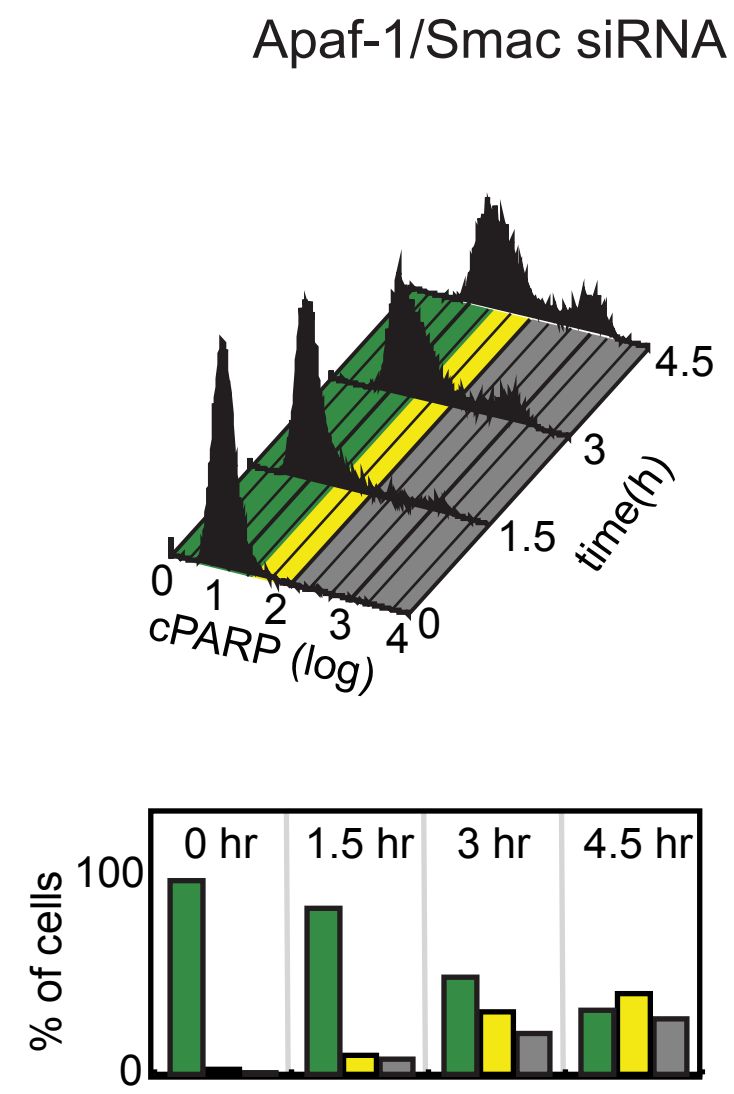
Apaf-1/Smac siRNA
0 1 2 3 4
cPARP (log)
0 1.5 3 4.5
time(h)
% of cells
100
0
0 hr
1.5 hr
3 hr
4.5 hr

D.

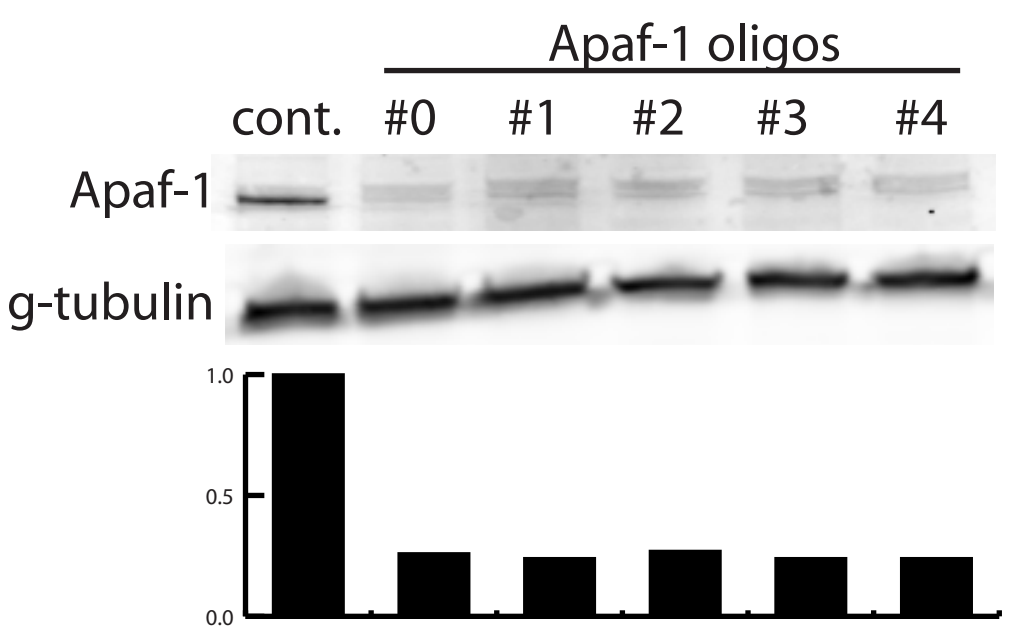
Apaf-1 oligos
cont.
#0
#1
#2
#3
#4
Apaf-1
g-tubulin
1.0
0.5
0.0
