# Supplementary material for: Modeling a Snap-Action, Variable-Delay Switch Controlling Extrinsic Cell Death
Source: PLoS Biol. 2008 Dec 2;6(12):e299. doi: 10.1371/journal.pbio.0060299 (PMC2592357; doi:10.1371/journal.pbio.0060299)
Supplement: Supplementary file 8 [file pbio.0060299.sg008.pdf]

A

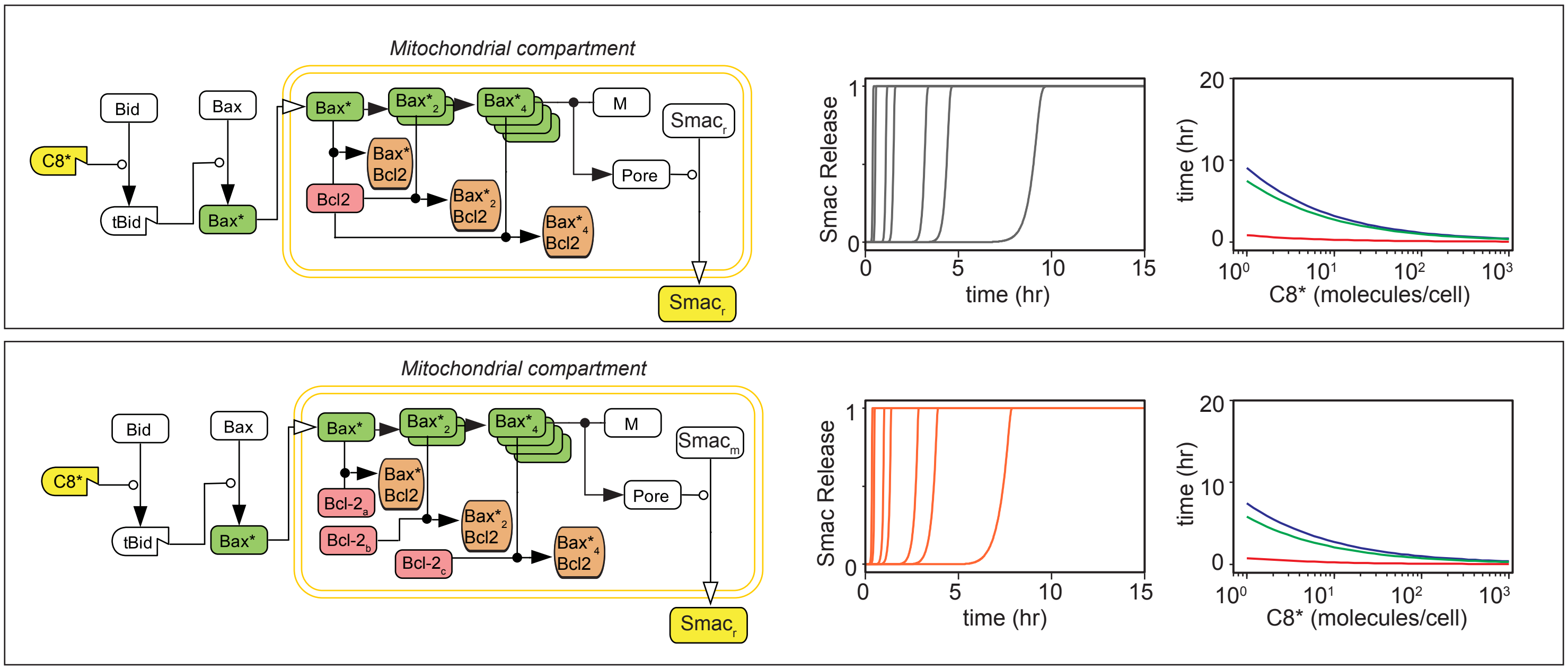
Mitochondrial compartment
Bid
Bax
C8*
tBid
Bax*
Bax*2
Bax*4
M
Smacr
Pore
Bcl2
Bax* Bcl2
Bax*2 Bcl2
Bax*4 Bcl2
Smacr
Smac Release
time (hr)
C8* (molecules/cell)
Mitochondrial compartment
Smacm
Bcl-2a
Bcl-2b
Bcl-2c

B

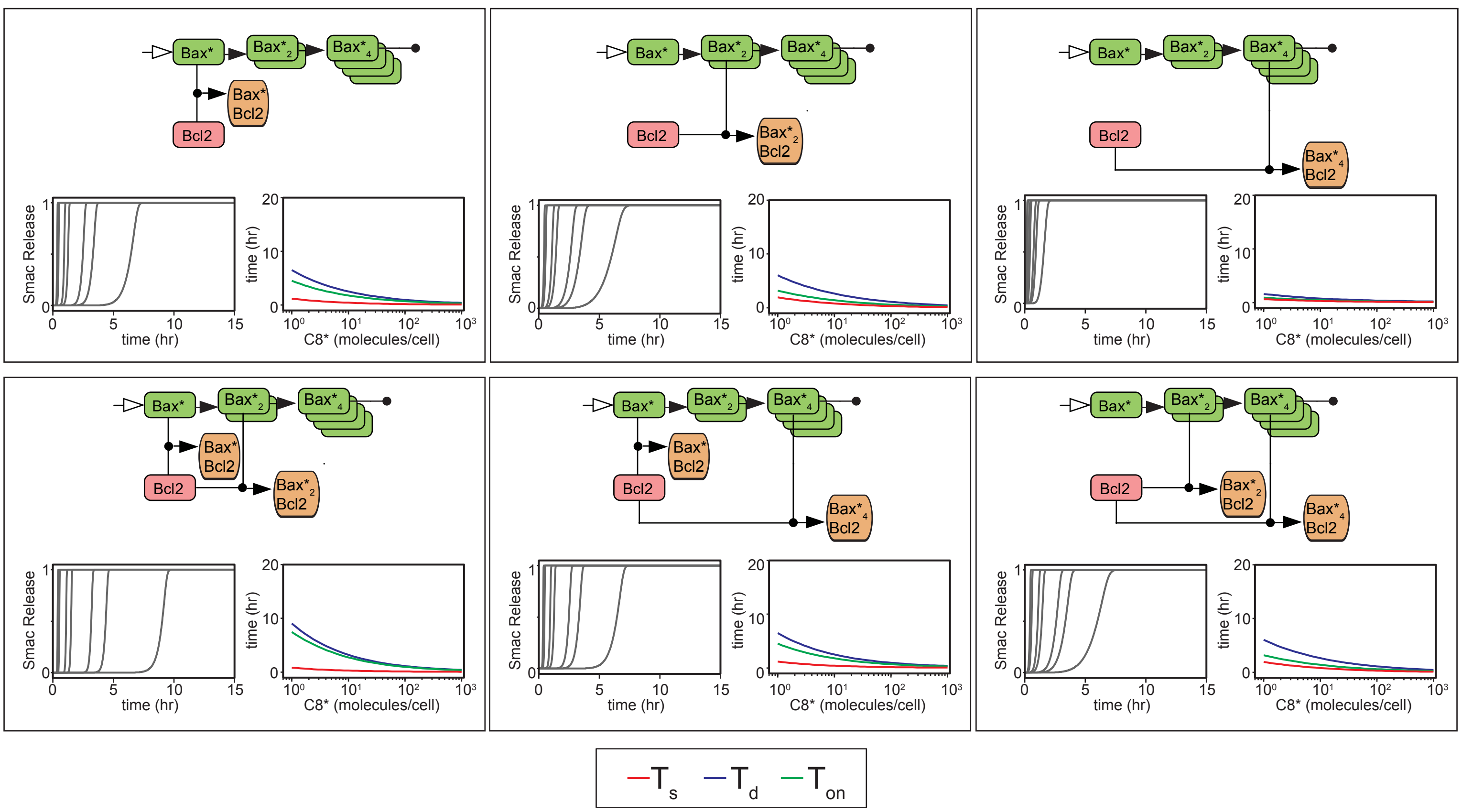
Bax*
Bax*2
Bax*4
Bcl2
Bax* Bcl2
Bax*2 Bcl2
Bax*4 Bcl2
Smac Release
time (hr)
C8* (molecules/cell)
Ts
Td
Ton
