# Supplementary material for: Modeling a Snap-Action, Variable-Delay Switch Controlling Extrinsic Cell Death
Source: PLoS Biol. 2008 Dec 2;6(12):e299. doi: 10.1371/journal.pbio.0060299 (PMC2592357; doi:10.1371/journal.pbio.0060299)
Supplement: Supplementary file 9 [file pbio.0060299.st001.pdf]

Table S1. List of Species in EARM v1.0

Species	Description (all species are located in cellular compartment (CC) unless otherwise stated.)
L	death ligand, such as TRAIL and TNF
R	inactive receptor complex
R^*	active receptor complex
$flip$	binds to active receptor acting as an inhibitor
$X : Y = Y : X$	complex formed by species X and Y, for example, $L : R$ is the ligand-receptor complex
$C8$	procaspase-8 and procaspase-10, inactive form of caspase-8 and caspase-10
$C8^*$	cleaved caspase-8 and caspase-10, active form of caspase-8 and caspase-10
Bar	binds to $C8^*$ and acts as an inhibitor
$C3$	procaspase-3 and procaspase-7, inactive form of caspase-3 and caspase-7
$C3^*$	cleaved caspase-3 and caspase-7, active form of caspase-3 and caspase-7
$C6$	procaspase-6, inactive form of caspase-6
$C6^*$	cleaved caspase-6, active form of caspase-6
$XIAP$	X-linked Inhibitor of Apoptosis (XIAP) in the cell
$PARP$	DNA damage repair enzyme, here represents all substrates of $C3^*$
$cPARP$	cleaved $PARP$, measure of single cell death
Bid	substrate of cleaved caspase-8, inactive form
$tBid$	cleaved Bid, active form of Bid
$Bcl2_c$	represents the family of antiapoptotic Bcl-2 proteins in the cellular compartment (CC), it binds to $tBid$ and acts as in inhibitor
Bax	substrate of $tBid$, inactive form
Bax^*	active form of Bax
Bax^*_m	Bax^* in the mitochondrial compartment (MC)
Bax_2	represents $Bax^*_m : Bax^*_m$ in the MC
Bax_4	represents $Bax_2 : Bax_2$ in the MC
$Bcl2$	represents all antiapoptotic Bcl-2 proteins in the MC
M	the number of unoccupied Bax_4 binding sites on outer membrane of the mitochondria, in MC
M^*	the number of pores Bax_4 created on the outer membrane of the mitochondria, in MC
CyC_m	cyotochrome c inside the mitochondria, in MC
CyC_r	cytochrome c released from the mitochondria, but remaining in the MC
CyC	cytochrome c in the CC
$Smac_m$	Smac/Diablo inside the mitochondria, in MC
$Smac_r$	Smac/Diablo released from the mitochondria, but remaining in the MC
$Smac$	Smac/Diablo in the CC
$Apaf$	Apoptosis Activating Factor (Apaf-1), substrate of CyC, inactive form
$Apaf^*$	active form of Apaf-1
$C9$	inactive form of procaspase-9
$Apop$	the apoptosome, which is the complex $Apaf^* : C9$
$C3^*_{Ub}$	$C3^*$ ubiquinated and targeted for degradation, assumed inactive
