# Supplementary material for: Modeling a Snap-Action, Variable-Delay Switch Controlling Extrinsic Cell Death
Source: PLoS Biol. 2008 Dec 2;6(12):e299. doi: 10.1371/journal.pbio.0060299 (PMC2592357; doi:10.1371/journal.pbio.0060299)
Supplement: Supplementary file 10 [file pbio.0060299.st002.pdf]

Table S2. EARM v1.0 Biochemical Equations

Reaction	Ref
$L + R \underset{k_{-1}}{\overset{k_1}{\rightleftharpoons}} L:R \xrightarrow{\kappa_1} R^*$	(1)
$R^* + flip \underset{k_{-2}}{\overset{k_2}{\rightleftharpoons}} R^*:flip$	(2)
$R^* + C8 \underset{k_{-3}}{\overset{k_3}{\rightleftharpoons}} R^*:C8 \xrightarrow{\kappa_3} R^* + C8^*$	(3)
$C8^* + Bar \underset{k_{-4}}{\overset{k_4}{\rightleftharpoons}} C8^*:Bar$	(4)
$C8^* + C3 \underset{k_{-5}}{\overset{k_5}{\rightleftharpoons}} C8^*:C3 \xrightarrow{\kappa_5} C8^* + C3^*$	(5)
$C3^* + C6 \underset{k_{-6}}{\overset{k_6}{\rightleftharpoons}} C3^*:C6 \xrightarrow{\kappa_6} C3^* + C6^*$	(6)
$C6^* + C8 \underset{k_{-7}}{\overset{k_7}{\rightleftharpoons}} C6^*:C8 \xrightarrow{\kappa_7} C6^* + C8^*$	(7)
$C3^* + XIAP \underset{k_{-8}}{\overset{k_8}{\rightleftharpoons}} C3^*:XIAP \xrightarrow{\kappa_8} C3^*_{Ub} + XIAP$	(8,9)
$C3^* + PARP \underset{k_{-9}}{\overset{k_9}{\rightleftharpoons}} C3^*:PARP \xrightarrow{\kappa_9} C3^* + cPARP$	(10)
$C8^* + Bid \underset{k_{-10}}{\overset{k_{10}}{\rightleftharpoons}} C8^*:Bid \xrightarrow{\kappa_{10}} C8^* + tBid$	(11,12)
$Bid + Bcl2_c \underset{k_{-11}}{\overset{k_{11}}{\rightleftharpoons}} Bid:Bcl2_c$	(13)
$tBid + Bax \underset{k_{-12}}{\overset{k_{12}}{\rightleftharpoons}} tBid:Bax \xrightarrow{\kappa_{12}} tBid + Bax^*$	(14)
$Bax^* \underset{k_{-13}}{\overset{k_{13}}{\rightleftharpoons}} Bax^*_m$	(14)
$Bax^*_m + Bcl2 \underset{k_{-14}}{\overset{k_{14}}{\rightleftharpoons}} Bax^*_m:Bcl2$	(15)
$Bax^*_m + Bax^*_m \underset{k_{-15}}{\overset{k_{15}}{\rightleftharpoons}} Bax_2$	(16)
$Bax_2 + Bcl2 \underset{k_{-16}}{\overset{k_{16}}{\rightleftharpoons}} Bax_2:Bcl2$	
$Bax_2 + Bax_2 \underset{k_{-17}}{\overset{k_{17}}{\rightleftharpoons}} Bax_4$	(17)
$Bax_4 + Bcl2 \underset{k_{-18}}{\overset{k_{18}}{\rightleftharpoons}} Bax_4:Bcl2$	
$Bax_4 + M \underset{k_{-19}}{\overset{k_{19}}{\rightleftharpoons}} Bax_4:M \xrightarrow{\kappa_{19}} M^*$	(17)
$M^* + CyC_m \underset{k_{-20}}{\overset{k_{20}}{\rightleftharpoons}} M^*:CyC_m \xrightarrow{\kappa_{20}} M^* + CyC_r$	(18)
$M^* + Smac_m \underset{k_{-21}}{\overset{k_{21}}{\rightleftharpoons}} M^*:Smac_m \xrightarrow{\kappa_{21}} M^* + Smac_r$	(19,20)
$CyC_r \underset{k_{-22}}{\overset{k_{22}}{\rightleftharpoons}} CyC$	
$CyC + Apaf \underset{k_{-23}}{\overset{k_{23}}{\rightleftharpoons}} CyC:Apaf \xrightarrow{\kappa_{23}} CyC + Apaf^*$	(21)
$Apaf^* + C9 \underset{k_{-24}}{\overset{k_{24}}{\rightleftharpoons}} Apop$	(18)
$Apop + C3 \underset{k_{-25}}{\overset{k_{25}}{\rightleftharpoons}} Apop:C3 \xrightarrow{\kappa_{25}} Apop + C3^*$	(18)
$Smac_r \underset{k_{-26}}{\overset{k_{26}}{\rightleftharpoons}} Smac$	
$Apop + XIAP \underset{k_{-27}}{\overset{k_{27}}{\rightleftharpoons}} Apop:XIAP$	(22,23)
$Smac + XIAP \underset{k_{-28}}{\overset{k_{28}}{\rightleftharpoons}} Smac:XIAP$	(19,20)

References

1. Chaudhary, P.M. et al. Death receptor 5, a new member of the TNFR family, and DR4 induce FADD-dependent apoptosis and activate the NF-kappaB pathway. *Immunity* **7**, 821-30 (1997).

2. Scaffidi, C., Schmitz, I., Krammer, P.H. & Peter, M.E. The role of c-FLIP in modulation of CD95-induced apoptosis. *J Biol Chem* **274**, 1541-8. (1999).

3. Medema, J.P. et al. FLICE is activated by association with the CD95 death-inducing signaling complex (DISC). *Embo J* **16**, 2794-804. (1997).

4. Stegh, A.H. et al. Inactivation of caspase-8 on mitochondria of Bcl-xL-expressing MCF7-Fas cells: role for the bifunctional apoptosis regulator protein. *J Biol Chem* **277**, 4351-60. (2002).

5. Stennicke, H.R. et al. Pro-caspase-3 is a major physiologic target of caspase-8. *J Biol Chem* **273**, 27084-90 (1998).

6. Slee, E.A. et al. Ordering the cytochrome c-initiated caspase cascade: hierarchical activation of caspases-2, -3, -6, -7, -8, and -10 in a caspase-9-dependent manner. *J Cell Biol* **144**, 281-92. (1999).

7. Cowling, V. & Downward, J. Caspase-6 is the direct activator of caspase-8 in the cytochrome c-induced apoptosis pathway: absolute requirement for removal of caspase-6 prodomain. *Cell Death Differ* **9**, 1046-56 (2002).

8. Deveraux, Q.L., Takahashi, R., Salvesen, G.S. & Reed, J.C. X-linked IAP is a direct inhibitor of cell-death proteases. *Nature* **388**, 300-4. (1997).

9. Suzuki, Y., Nakabayashi, Y. & Takahashi, R. Ubiquitin-protein ligase activity of X-linked inhibitor of apoptosis protein promotes proteasomal degradation of caspase-3 and enhances its anti-apoptotic effect in Fas-induced cell death. *Proc Natl Acad Sci U S A* **98**, 8662-7 (2001).

10. Tewari, M. et al. Yama/CPP32 beta, a mammalian homolog of CED-3, is a CrmA-inhibitable protease that cleaves the death substrate poly(ADP-ribose) polymerase. *Cell* **81**, 801-9 (1995).

11. Luo, X., Budihardjo, I., Zou, H., Slaughter, C. & Wang, X. Bid, a Bcl2 interacting protein, mediates cytochrome c release from mitochondria in response to activation of cell surface death receptors. *Cell* **94**, 481-90. (1998).

12. Li, H., Zhu, H., Xu, C.J. & Yuan, J. Cleavage of BID by caspase 8 mediates the mitochondrial damage in the Fas pathway of apoptosis. *Cell* **94**, 491-501. (1998).

13. Wang, K., Yin, X.M., Chao, D.T., Milliman, C.L. & Korsmeyer, S.J. BID: a novel BH3 domain-only death agonist. *Genes Dev* **10**, 2859-69 (1996).

14. Eskes, R., Desagher, S., Antonsson, B. & Martinou, J.C. Bid induces the oligomerization and insertion of Bax into the outer mitochondrial membrane. *Mol Cell Biol* **20**, 929-35. (2000).

15. Oltvai, Z.N., Milliman, C.L. & Korsmeyer, S.J. Bcl-2 heterodimerizes in vivo with a conserved homolog, Bax, that accelerates programmed cell death. *Cell* **74**, 609-19 (1993).

16. Mikhailov, V. et al. Bcl-2 prevents Bax oligomerization in the mitochondrial outer membrane. *J Biol Chem* **276**, 18361-74 (2001).

17. Kuwana, T. et al. Bid, Bax, and lipids cooperate to form supramolecular openings in the outer mitochondrial membrane. *Cell* **111**, 331-42 (2002).

18. Li, P. et al. Cytochrome c and dATP-dependent formation of Apaf-1/caspase-9 complex initiates an apoptotic protease cascade. *Cell* **91**, 479-89. (1997).

19. Verhagen, A.M. et al. Identification of DIABLO, a mammalian protein that promotes apoptosis by binding to and antagonizing IAP proteins. *Cell* **102**, 43-53. (2000).

20. Du, C., Fang, M., Li, Y., Li, L. & Wang, X. Smac, a mitochondrial protein that promotes cytochrome c-dependent caspase activation by eliminating IAP inhibition. *Cell* **102**, 33-42. (2000).

21. Kim, H.E., Du, F., Fang, M. & Wang, X. Formation of apoptosome is initiated by cytochrome c-induced dATP hydrolysis and subsequent nucleotide exchange on Apaf-1. *Proc Natl Acad Sci U S A* **102**, 17545-50 (2005).

22. Twiddy, D. et al. Pro-apoptotic proteins released from the mitochondria regulate the protein composition and caspase-processing activity of the native Apaf-1/caspase-9 apoptosome complex. *J Biol Chem* **279**, 19665-82 (2004).

23. Hill, M.M., Adrain, C., Duriez, P.J., Creagh, E.M. & Martin, S.J. Analysis of the composition, assembly kinetics and activity of native Apaf-1 apoptosomes. *Embo J* **23**, 2134-45 (2004).
