# Supplementary material for: Modeling a Snap-Action, Variable-Delay Switch Controlling Extrinsic Cell Death
Source: PLoS Biol. 2008 Dec 2;6(12):e299. doi: 10.1371/journal.pbio.0060299 (PMC2592357; doi:10.1371/journal.pbio.0060299)
Supplement: Supplementary file 11 [file pbio.0060299.st003.pdf]

Table S3. EARM v1.0 Rate Constant Values

i	k_i $((\#/CC)^{-1}\cdot sec^{-1})$	k_i $((M)^{-1}\cdot sec^{-1})$	k_{-i} (sec^{-1})	κ_i (sec^{-1})
1	4×10^{-7}	2.4×10^5	10^{-3}	10^{-5}
2	10^{-6}	6×10^5	10^{-3}	
3	10^{-6}	6×10^5	10^{-3}	1
4	10^{-6}	6×10^5	10^{-3}	
5	10^{-7}	6×10^4	10^{-3}	1
6	10^{-6}	6×10^5	10^{-3}	1
7	3×10^{-8}	1.8×10^4	10^{-3}	1
8	2×10^{-6}	1.2×10^6	10^{-3}	0.1
9	10^{-6}	6×10^5	10^{-2}	1
10	10^{-7}	6×10^4	10^{-3}	1
11	10^{-6}	6×10^5	10^{-3}	
12	10^{-7}	6×10^4	10^{-3}	1
13	10^{-2} (sec^{-1})		10^{-2}	
14	10^{-6} $((\#/MC)^{-1}\cdot sec^{-1})$	6×10^5	10^{-3}	
15	10^{-6} $((\#/MC)^{-1}\cdot sec^{-1})$	6×10^5	10^{-3}	
16	10^{-6} $((\#/MC)^{-1}\cdot sec^{-1})$	6×10^5	10^{-3}	
17	10^{-6} $((\#/MC)^{-1}\cdot sec^{-1})$	6×10^5	10^{-3}	
18	10^{-6} $((\#/MC)^{-1}\cdot sec^{-1})$	6×10^5	10^{-3}	
19	10^{-6} $((\#/MC)^{-1}\cdot sec^{-1})$	6×10^5	10^{-3}	1
20	2×10^{-6} $((\#/MC)^{-1}\cdot sec^{-1})$	1.2×10^6	10^{-3}	10
21	2×10^{-6} $((\#/MC)^{-1}\cdot sec^{-1})$	1.2×10^6	10^{-3}	10
22	10^{-2} (sec^{-1})		10^{-2}	
23	5×10^{-7}	3×10^5	10^{-3}	1
24	5×10^{-8}	3×10^4	10^{-3}	
25	5×10^{-9}	3×10^3	10^{-3}	1
26	10^{-2} (sec^{-1})		10^{-2}	
27	2×10^{-6}	1.2×10^6	10^{-3}	
28	7×10^{-6}	4.2×10^6	10^{-3}	
