# Supplementary material for: Modeling a Snap-Action, Variable-Delay Switch Controlling Extrinsic Cell Death
Source: PLoS Biol. 2008 Dec 2;6(12):e299. doi: 10.1371/journal.pbio.0060299 (PMC2592357; doi:10.1371/journal.pbio.0060299)
Supplement: Supplementary file 12 [file pbio.0060299.st004.pdf]

Table S4. References for Rate Constants

Enzymatic Reaction	Km (#/cell) or (M)	k_{cat} (sec^{-1})	k_{cat}/Km (cell/#/sec) or (1/M/sec)	Ref	substrate
$C8^*$					
Garcia-Calvo *et.al.* 1999	$4.2 \times 10^7/7.0 \times 10^{-5}$	0.37	$8.8 \times 10^{-9}/5300$	(1)	LETD
Stennicke *et.al.* 1998			$1.5 \times 10^{-6}/9.0 \times 10^5$	(2)	caspase-3
Stennicke *et.al.* 2000	$1.1 \times 10^7/1.8 \times 10^{-5}$	0.69	$6.4 \times 10^{-8}/3.8 \times 10^4$	(3)	DEVDG
$C3^*$					
Bose *et.al.* 2003	$1.3 \times 10^6/2.2 \times 10^{-6}$	0.4	$3.0 \times 10^{-7}/1.8 \times 10^5$	(4)	DEVD
Casciola-Rosen *et.al.* 1996			$8.6 \times 10^{-6}/5.2 \times 10^6$	(5)	PARP
Casciola-Rosen *et.al.* 1996			$1.2 \times 10^{-5}/7.2 \times 10^6$	(5)	DNA-PK
Casciola-Rosen *et.al.* 1996			$3.8 \times 10^{-6}/2.3 \times 10^6$	(5)	UI-70K
Garcia-Calvo *et.al.* 1999	$6.0 \times 10^6/1.0 \times 10^{-5}$	14	$2.3 \times 10^{-6}/1.4 \times 10^6$	(1)	DEVD
Margolin *et.al.* 1997	$6.0 \times 10^6/1.0 \times 10^{-5}$	23	$3.8 \times 10^{-6}/2.3 \times 10^6$	(6)	PARP
Stennicke *et.al.* 2000	$7.8 \times 10^6/1.3 \times 10^{-5}$	2.5	$3.2 \times 10^{-7}/1.9 \times 10^5$	(3)	DEVDG
Talanian *et.al.* 1997	$6.6 \times 10^6/1.1 \times 10^{-5}$	2.4	$3.6 \times 10^{-7}/2.2 \times 10^5$	(7)	DEVD
$C6^*$					
Garcia-Calvo *et.al.* 1999	$1.0 \times 10^8/1.7 \times 10^{-4}$			(1)	VEHD
$C9$					
Garcia-Calvo *et.al.* 1999	$4.7 \times 10^8/7.8 \times 10^{-4}$	0.1	$2.6 \times 10^{-10}/160$	(1)	LEHD
Talanian *et.al.* 1997	$1.8 \times 10^7/3.0 \times 10^{-5}$	5	$2.7 \times 10^{-7}/1.6 \times 10^5$	(7)	VEID

Binding Reaction	k_{on} ($(\#/cell)^{-1} \cdot sec^{-1}$) or ($M^{-1} \cdot sec^{-1}$)	k_{off} (sec^{-1})	Kd (#/cell) or (M)	Ref	protein fragments
$XIAP : C3^*/7^*$					
Huang *et.al* 2003	$3.5 \times 10^{-6}/2.1 \times 10^6$	0.0021	$600/1 \times 10^{-9}$	(8)	caspase-7/linkerBIR2
Riedl *et.al* 2001	$4.2 \times 10^{-6}/2.5 \times 10^6$	0.0024	$570/9.6 \times 10^{-10}$	(9)	caspase-3/BIR2
Riedl *et.al* 2001	$2.3 \times 10^{-5}/1.4 \times 10^7$	0.0014	$61/1 \times 10^{-10}$	(9)	caspase-7/BIR2
$XIAP : Casp9$					
Sun *et.al* 2000			$6700/1.1 \times 10^{-8}$	(10)	BIR2-RF(aa 241-497) of XIAP
$Smac : XIAP$					
Hung *et.al* 2003	$1.2 \times 10^{-5}/7.2 \times 10^6$	0.0022	$180/3 \times 10^{-10}$	(8)	Smac/linker_BIR2_BIR3

References

1. Garcia-Calvo, M. et al. Purification and catalytic properties of human caspase family members. *Cell Death Differ* **6**, 362-9 (1999).

2. Stennicke, H.R. et al. Pro-caspase-3 is a major physiologic target of caspase-8. *J Biol Chem* **273**, 27084-90 (1998).

3. Stennicke, H.R., Renatus, M., Meldal, M. & Salvesen, G.S. Internally quenched fluorescent peptide substrates disclose the subsite preferences of human caspases 1, 3, 6, 7 and 8. *Biochem J* **350 Pt 2**, 563-8 (2000).

4. Bose, K., Pop, C., Feeney, B. & Clark, A.C. An uncleavable procaspase-3 mutant has a lower catalytic efficiency but an active site similar to that of mature caspase-3. *Biochemistry* **42**, 12298-310 (2003).

5. Casciola-Rosen, L. et al. Apopain/CPP32 cleaves proteins that are essential for cellular repair: a fundamental principle of apoptotic death. *J Exp Med* **183**, 1957-64 (1996).

6. Margolin, N. et al. Substrate and inhibitor specificity of interleukin-1 beta-converting enzyme and related caspases. *J Biol Chem* **272**, 7223-8 (1997).

7. Talanian, R.V. et al. Substrate specificities of caspase family proteases. *J Biol Chem* **272**, 9677-82 (1997).

8. Huang, Y., Rich, R.L., Myszka, D.G. & Wu, H. Requirement of both the second and third BIR domains for the relief of X-linked inhibitor of apoptosis protein (XIAP)-mediated caspase inhibition by Smac. *J Biol Chem* **278**, 49517-22 (2003).

9. Riedl, S.J. et al. Structural basis for the inhibition of caspase-3 by XIAP. *Cell* **104**, 791-800 (2001).

10. Sun, C. et al. NMR structure and mutagenesis of the third Bir domain of the inhibitor of apoptosis protein XIAP. *J Biol Chem* **275**, 33777-81 (2000).
