# Supplementary material for: Modeling a Snap-Action, Variable-Delay Switch Controlling Extrinsic Cell Death
Source: PLoS Biol. 2008 Dec 2;6(12):e299. doi: 10.1371/journal.pbio.0060299 (PMC2592357; doi:10.1371/journal.pbio.0060299)
Supplement: Supplementary file 13 [file pbio.0060299.st005.pdf]

Table S5. Variable Definitions and Initial Conditions for EARM v1.0

variable	state	model IC (#/CC) or (M)	ref
x_1	$[L]$	$3 \times 10^3/5.1 \times 10^{-9} = 50$ ng/ml	
x_2	$[R]$ WT	$2 \times 10^2/3.4 \times 10^{-10}$	
	$[R]$ siRNA-treated	$10^5/1.7 \times 10^{-7}$	
x_3	$[L:R]$	0	
x_4	$[R^*]$	0	
x_5	$[flip]$	$10^2/1.7 \times 10^{-10}$	
x_6	$[flip:R^*]$	0	
x_7	$[C8]$	$2 \times 10^4/3.4 \times 10^{-8}$	(1)
x_8	$[C8:R^*]$	0	
x_9	$[C8^*]$	0	
x_{10}	$[Bar]$	$10^3/1.7 \times 10^{-9}$	
x_{11}	$[C8^*:Bar]$	0	
x_{12}	$[C3]$	$10^4/1.7 \times 10^{-8}$	(1, 2 ,3)
x_{13}	$[C8^*:C3]$	0	
x_{14}	$[C3^*]$	0	
x_{15}	$[C6]$	$10^4/1.7 \times 10^{-8}$	
x_{16}	$[C3^*:C6]$	0	
x_{17}	$[C6^*]$	0	
x_{18}	$[C6^*:C8]$	0	
x_{19}	$[XIAP]$	$10^5/1.7 \times 10^{-7}$	(3)
x_{20}	$[XIAP:C3^*]$	0	
x_{21}	$[PARP]$	$10^6/1.7 \times 10^{-6}$	
x_{22}	$[C3^*:PARP]$	0	
x_{23}	$[cPARP]$	0	
x_{24}	$[Bid]$	4×10^4	
x_{25}	$[C8^*:Bid]$	0	
x_{26}	$[tBid]$	0	
x_{27}	$[Bcl2_c]$	$2 \times 10^4/3.4 \times 10^{-8}$	
x_{28}	$[tBid:Bcl2_c]$	0	
x_{29}	$[Bax]$	$10^5/1.7 \times 10^{-7}$	
x_{30}	$[tBid:Bax]$	0	
x_{31}	$[Bax^*]$	0	
x_{32}	$[Bax^*_m]$	0 (#/MC)	
x_{33}	$[Bcl2]$	2×10^4 (#/MC)/4.8×10^{-7}	
x_{34}	$[Bax^*_m:Bcl2]$	0 (#/MC)	
x_{35}	$[Bax_2]$	0 (#/MC)	
x_{36}	$[Bax_2:Bcl2]$	0 (#/MC)	
x_{37}	$[Bax_4]$	0 (#/MC)	
x_{38}	$[Bax_4:Bcl2]$	0 (#/MC)	
x_{39}	$[M]$	5×10^5 (#/MC)/1.2×10^{-5}	
x_{40}	$[Bax_4:M]$	0 (#/MC)	
x_{41}	$[M^*]$	0 (#/MC)	
x_{42}	$[CyC_m]$	5×10^5 (#/MC)/1.2×10^{-5}	(4)
x_{43}	$[M^*:CyC_m]$	0 (#/MC)	
x_{44}	$[CyC_r]$	0 (#/MC)	
x_{45}	$[Smac_m]$	10^5 (#/MC)/2.4×10^{-6}	
x_{46}	$[M^*:Smac_m]$	0 (#/MC)	
x_{47}	$[Smac_r]$	0 (#/MC)	
x_{48}	$[CyC]$	0	
x_{49}	$[Apaf]$	$10^5/1.7 \times 10^{-7}$	(3)
x_{50}	$[Apaf:CyC]$	0	
x_{51}	$[Apaf^*]$	0	
x_{52}	$[C9]$	$10^5/1.7 \times 10^{-7}$	(3)
x_{53}	$[Apop]$	0	
x_{54}	$[Apop:C3]$	0	
x_{55}	$[Smac]$	0	
x_{56}	$[Apop:XIAP]$	0	
x_{57}	$[Smac:XIAP]$	0	
x_{58}	$[C3^*_{Ub}]$	0	

References

1. Eissing, T. et al. Bistability analyses of a caspase activation model for receptor-induced apoptosis. *J Biol Chem* **279**, 36892-7 (2004).

2. Stennicke, H.R. et al. Pro-caspase-3 is a major physiologic target of caspase-8. *J Biol Chem* **273**, 27084-90 (1998).

3. Rehm, M., Huber, H.J., Dussmann, H. & Prehn, J.H. Systems analysis of effector caspase activation and its control by X-linked inhibitor of apoptosis protein. *Embo J* **25**, 4338-49 (2006).

4. Waterhouse, N.J. et al. Cytochrome c maintains mitochondrial transmembrane potential and ATP generation after outer mitochondrial membrane permeabilization during the apoptotic process. *J Cell Biol* **153**, 319-28 (2001).
