# Supplementary material for: Modeling a Snap-Action, Variable-Delay Switch Controlling Extrinsic Cell Death
Source: PLoS Biol. 2008 Dec 2;6(12):e299. doi: 10.1371/journal.pbio.0060299 (PMC2592357; doi:10.1371/journal.pbio.0060299)
Supplement: Supplementary file 14 [file pbio.0060299.st006.pdf]

Table S6. EARM v1.0 Equations

$$
\begin{aligned}
\dot{x}_1 &= -k_1 x_1 x_2 + k_{-1} x_3\\
\dot{x}_2 &= -k_1 x_1 x_2 + k_{-1} x_3\\
\dot{x}_3 &= k_1 x_1 x_2 - k_{-1} x_3 - \kappa_1 x_3\\
\dot{x}_4 &= \kappa_1 x_3 - k_2 x_4 x_5 + k_{-2} x_6 + \ldots\\
&\quad -k_3 x_4 x_7 + k_{-3} x_8 + \kappa_3 x_8\\
\dot{x}_5 &= -k_2 x_4 x_5 + k_{-2} x_6\\
\dot{x}_6 &= k_2 x_4 x_5 - k_{-2} x_6\\
\dot{x}_7 &= -k_3 x_4 x_7 + k_{-3} x_8 + \ldots\\
&\quad -k_7 x_7 x_{17} + k_{-7} x_{18}\\
\dot{x}_8 &= k_3 x_4 x_7 - k_{-3} x_8 - \kappa_3 x_8\\
\dot{x}_9 &= \kappa_3 x_8 - k_4 x_9 x_{10} + k_{-4} x_{11} + \ldots\\
&\quad -k_5 x_9 x_{12} + k_{-5} x_{13} + \kappa_5 x_{13} + \ldots\\
&\quad +\kappa_7 x_{18} + \ldots\\
&\quad -k_{10} x_9 x_{24} + k_{-10} x_{25} + \kappa_{10} x_{25}\\
\dot{x}_{10} &= -k_4 x_9 x_{10} + k_{-4} x_{11}\\
\dot{x}_{11} &= k_4 x_9 x_{10} - k_{-4} x_{11}\\
\dot{x}_{12} &= -k_5 x_9 x_{12} + k_{-5} x_{13} + \ldots\\
&\quad -k_{25} x_{12} x_{53} + k_{-25} x_{54}\\
\dot{x}_{13} &= k_5 x_9 x_{12} - k_{-5} x_{13} - \kappa_5 x_{13}\\
\dot{x}_{14} &= \kappa_5 x_{13} + \kappa_{25} x_{54} + \ldots\\
&\quad -k_6 x_{14} x_{15} + k_{-6} x_{16} + \kappa_6 x_{16} + \ldots\\
&\quad -k_8 x_{14} x_{19} + k_{-8} x_{20} + \ldots\\
&\quad -k_9 x_{14} x_{21} + k_{-9} x_{22} + \kappa_9 x_{22}\\
\dot{x}_{15} &= -k_6 x_{14} x_{15} + k_{-6} x_{16}\\
\dot{x}_{16} &= k_6 x_{14} x_{15} - k_{-6} x_{16} - \kappa_6 x_{16}\\
\dot{x}_{17} &= \kappa_6 x_{16} + \ldots\\
&\quad -k_7 x_7 x_{17} + k_{-7} x_{18} + \kappa_7 x_{18}\\
\dot{x}_{18} &= k_7 x_7 x_{17} - k_{-7} x_{18} - \kappa_7 x_{18}\\
\dot{x}_{19} &= -k_8 x_{14} x_{19} + k_{-8} x_{20} + \kappa_8 x_{20} + \ldots\\
&\quad -k_{27} x_{19} x_{53} + k_{-27} x_{56} + \ldots\\
&\quad -k_{28} x_{19} x_{55} + k_{-28} x_{57}\\
\dot{x}_{20} &= k_8 x_{14} x_{19} - k_{-8} x_{20} - \kappa_8 x_{20}\\
\dot{x}_{21} &= -k_9 x_{14} x_{21} + k_{-9} x_{22}\\
\dot{x}_{22} &= k_9 x_{14} x_{21} - k_{-9} x_{22} - \kappa_9 x_{22}\\
\dot{x}_{23} &= \kappa_9 x_{22}\\
\dot{x}_{24} &= -k_{10} x_9 x_{24} + k_{-10} x_{25}\\
\dot{x}_{25} &= k_{10} x_9 x_{24} - k_{-10} x_{25} - \kappa_{10} x_{25}\\
\dot{x}_{26} &= \kappa_{10} x_{25} - k_{11} x_{26} x_{27} + k_{-11} x_{28} + \ldots\\
&\quad -k_{12} x_{26} x_{29} + k_{-12} x_{30} + \kappa_{12} x_{30}\\
\dot{x}_{27} &= -k_{11} x_{26} x_{27} + k_{-11} x_{28}\\
\dot{x}_{28} &= k_{11} x_{26} x_{27} - k_{-11} x_{28}\\
\dot{x}_{29} &= -k_{12} x_{26} x_{29} + k_{-12} x_{30}\\
\dot{x}_{30} &= k_{12} x_{26} x_{29} - k_{-12} x_{30} - \kappa_{12} x_{30}\\
\dot{x}_{31} &= \kappa_{12} x_{30} - k_{13} x_{31} + k_{-13} x_{32}\\
\dot{x}_{32} &= k_{13} x_{31} - k_{-13} x_{32} + \ldots\\
&\quad -\frac{1}{v} k_{14} x_{32} x_{33} + k_{-14} x_{34} + \ldots\\
&\quad -\frac{2}{v} k_{15} x_{32}^2 + 2k_{-15} x_{35}
\end{aligned}
$$

$$
\begin{aligned}
\dot{x}_{33} &= -\frac{1}{v} k_{14} x_{32} x_{33} + k_{-14} x_{34} + \ldots\\
&\quad -\frac{1}{v} k_{16} x_{33} x_{35} + k_{-16} x_{36} + \ldots\\
&\quad -\frac{1}{v} k_{18} x_{33} x_{37} + k_{-18} x_{38}\\
\dot{x}_{34} &= \frac{1}{v} k_{14} x_{32} x_{33} - k_{-14} x_{34}\\
\dot{x}_{35} &= \frac{1}{v} k_{15} x_{32}^2 - k_{-15} x_{35} + \ldots\\
&\quad -\frac{1}{v} k_{16} x_{33} x_{35} + k_{-16} x_{36} + \ldots\\
&\quad -\frac{2}{v} k_{17} x_{35}^2 + 2k_{-17} x_{37}\\
\dot{x}_{36} &= \frac{1}{v} k_{16} x_{33} x_{35} - k_{-16} x_{36}\\
\dot{x}_{37} &= \frac{1}{v} k_{17} x_{35}^2 - k_{-17} x_{37} + \ldots\\
&\quad -\frac{1}{v} k_{18} x_{33} x_{37} + k_{-18} x_{38} + \ldots\\
&\quad -\frac{1}{v} k_{19} x_{39} x_{37} + k_{-19} x_{40}\\
\dot{x}_{38} &= \frac{1}{v} k_{18} x_{33} x_{37} - k_{-18} x_{38}\\
\dot{x}_{39} &= -\frac{1}{v} k_{19} x_{39} x_{37} + k_{-19} x_{40}\\
\dot{x}_{40} &= \frac{1}{v} k_{19} x_{39} x_{37} - k_{-19} x_{40} - \kappa_{19} x_{40}\\
\dot{x}_{41} &= \kappa_{19} x_{40} + \ldots\\
&\quad -\frac{1}{v} k_{20} x_{41} x_{42} + k_{-20} x_{43} + \kappa_{20} x_{43} + \ldots\\
&\quad -\frac{1}{v} k_{21} x_{41} x_{45} + k_{-21} x_{46} + \kappa_{21} x_{46}\\
\dot{x}_{42} &= -\frac{1}{v} k_{20} x_{41} x_{42} + k_{-20} x_{43}\\
\dot{x}_{43} &= \frac{1}{v} k_{20} x_{41} x_{42} - k_{-20} x_{43} - \kappa_{20} x_{43}\\
\dot{x}_{44} &= \kappa_{20} x_{43} - k_{22} x_{44} + k_{-22} x_{48}\\
\dot{x}_{45} &= -\frac{1}{v} k_{21} x_{41} x_{45} + k_{-21} x_{46}\\
\dot{x}_{46} &= \frac{1}{v} k_{21} x_{41} x_{45} - k_{-21} x_{46} - \kappa_{21} x_{46}\\
\dot{x}_{47} &= \kappa_{21} x_{46} - k_{26} x_{47} + k_{-26} x_{55}\\
\dot{x}_{48} &= k_{22} x_{44} - k_{-22} x_{48} + \ldots\\
&\quad -k_{23} x_{48} x_{49} + k_{-23} x_{50} + \kappa_{23} x_{50}\\
\dot{x}_{49} &= -k_{23} x_{48} x_{49} + k_{-23} x_{50}\\
\dot{x}_{50} &= k_{23} x_{48} x_{49} - k_{-23} x_{50} - \kappa_{23} x_{50}\\
\dot{x}_{51} &= \kappa_{23} x_{50} - k_{24} x_{51} x_{52} + k_{-24} x_{53}\\
\dot{x}_{52} &= -k_{24} x_{51} x_{52} + k_{-24} x_{53}\\
\dot{x}_{53} &= k_{24} x_{51} x_{52} - k_{-24} x_{53} + \ldots\\
&\quad -k_{25} x_{12} x_{53} + k_{-25} x_{54} + \kappa_{25} x_{54} + \ldots\\
&\quad -k_{27} x_{19} x_{53} + k_{-27} x_{56}\\
\dot{x}_{54} &= k_{25} x_{12} x_{53} - k_{-25} x_{54} - \kappa_{25} x_{54}\\
\dot{x}_{55} &= k_{26} x_{47} - k_{-26} x_{55} + \ldots\\
&\quad -k_{28} x_{19} x_{55} + k_{-28} x_{57}\\
\dot{x}_{56} &= k_{27} x_{19} x_{53} - k_{-27} x_{56}\\
\dot{x}_{57} &= k_{28} x_{19} x_{55} - k_{-28} x_{57}\\
\dot{x}_{58} &= \kappa_8 x_{20}
\end{aligned}
$$
