# Supplementary material for: Modeling a Snap-Action, Variable-Delay Switch Controlling Extrinsic Cell Death
Source: PLoS Biol. 2008 Dec 2;6(12):e299. doi: 10.1371/journal.pbio.0060299 (PMC2592357; doi:10.1371/journal.pbio.0060299)
Supplement: Supplementary file 15 [file pbio.0060299.st007.pdf]

Table S7: Sequences of siRNA Oligonucleotides

	Target sequence	Source
**Apaf-1**		
oligo1	aattggtgcacttttacgtga	[1]
oligo 2	aatgatggcattcctgttgtc	Designed
oligo 3	aacacggttggatcaggatga	Designed
oligo 4	nnacaactttctggcctatca	Designed
oligo 5	nnggtcgataatggaatgctt	Designed
**Caspase-8**		
oligo 1	aactaccagaaaggtatacct	[2]
**Bid**		
oligo 1	aagaatagaggcagattctga	[3]
**Smac**		
oligo 1	nngaccacatatgcgttgatt	[4]
oligo 2	nntgcgttgattgaagctatt	[4]
oligo 3	nnacttctctttaccgacaat	[4]
oligo 4	nngcagaagctgcatatcaaa	[4]
**XIAP**		
oligo 1	aaacttgtgtacctgcagaca	[4]
oligo 2	aaccgtgcggtgctttagttg	[4]
oligo 3	aaggcgacactttcctaattg	[4]
oligo 4	aaaatagtgccacgcagtcta	[4]
**controls**		
EGFP-1	aaagcagaagaacggcatcaa	[4]
EGFP-2	aagctcaagcttcgaattctg	[4]
siControl non-targeting 1	proprietary	Dharmacon
siControl non-targeting 3	proprietary	Dharmacon

References

1. Lassus, P., Opitz-Araya, X. & Lazebnik, Y. Requirement for caspase-2 in stress-induced apoptosis before mitochondrial permeabilization. *Science* **297**, 1352-4 (2002).

2. Chun, H.J. et al. Pleiotropic defects in lymphocyte activation caused by caspase-8 mutations lead to human immunodeficiency. *Nature* **419**, 395-9 (2002).

3. Aza-Blanc, P. et al. Identification of modulators of TRAIL-induced apoptosis via RNAi-based phenotypic screening. *Mol Cell* **12**, 627-37 (2003).

4. Albeck, J.G. et al. Quantitative analysis of pathways controlling extrinsic apoptosis in single cells. *Mol Cell* **30**, 11-25 (2008).
